# Machine learning-guided discovery and design of non-hemolytic peptides

**DOI:** 10.1038/s41598-020-73644-6

**Published:** 2020-10-06

**Authors:** Fabien Plisson, Obed Ramírez-Sánchez, Cristina Martínez-Hernández

**Affiliations:** 1 CONACYT, Unidad de Genómica Avanzada, Laboratorio Nacional de Genómica para la Biodiversidad (Langebio), Centro de Investigación Y de Estudios Avanzados del IPN, 36824 Irapuato, Guanajuato Mexico; 2 Unidad de Genómica Avanzada, Laboratorio Nacional de Genómica para la Biodiversidad (Langebio), Centro de Investigación Y de Estudios Avanzados del IPN, 36824 Irapuato, Guanajuato Mexico

**Keywords:** Protein sequence analyses, Cheminformatics, Peptides, Data acquisition, Data mining, Data processing, Machine learning, Protein design, Statistical methods

## Abstract

Reducing hurdles to clinical trials without compromising the therapeutic promises of peptide candidates becomes an essential step in peptide-based drug design. Machine-learning models are cost-effective and time-saving strategies used to predict biological activities from primary sequences. Their limitations lie in the diversity of peptide sequences and biological information within these models. Additional outlier detection methods are needed to set the boundaries for reliable predictions; the applicability domain. Antimicrobial peptides (AMPs) constitute an extensive library of peptides offering promising avenues against antibiotic-resistant infections. Most AMPs present in clinical trials are administrated topically due to their hemolytic toxicity. Here we developed machine learning models and outlier detection methods that ensure robust predictions for the discovery of AMPs and the design of novel peptides with reduced hemolytic activity. Our best models, gradient boosting classifiers, predicted the hemolytic nature from any peptide sequence with 95–97% accuracy. Nearly 70% of AMPs were predicted as hemolytic peptides. Applying multivariate outlier detection models, we found that 273 AMPs (~ 9%) could not be predicted reliably. Our combined approach led to the discovery of 34 high-confidence non-hemolytic natural AMPs, the de novo design of 507 non-hemolytic peptides, and the guidelines for non-hemolytic peptide design.

## Introduction

Peptides play essential roles in human physiology targeting growth factors, ion channels, protein receptors or enzymes^1,2^. They exhibit a broad range of biological activities as antimicrobial^3^, antifungal^4^, antiviral^5^, antiparasitic^6^, insecticidal^7^ or anticancer agents^8^; all valuable starting points to treat human disorders and needs. Some demonstrate good pharmacokinetic properties, all considered desirable for treatments against cancer, immune disorders, cardiovascular diseases, gastrointestinal dysfunction, haemostasis and microbial infections. Despite these advantages, many peptides do not translate into clinics due to metabolic stability (or lack thereof), lability during storage, poor oral bioavailability and undesirable toxicities (cytotoxicity, immunotoxicity, hemotoxicity)^1,9^. To date, interest in peptide-based drugs is steadily increasing with 60 commercialised therapeutic peptides and, 150 are in clinical development^2^.

Reducing hurdles to preclinical and clinical trials without compromising therapeutic profiles of candidates becomes an essential step in drug design. Peptide-based drug design operates traditionally through a series of modifications, i.e. alanine scan, single mutations, truncations, deletions leading to an extensive library of peptide analogues^1^. Peptides are then evaluated through biological assays in an iterative process to identify critical residues, also known as structure–activity relationship studies. Such biological assays may be straightforward to carry out for few peptides; they hit a bottleneck with more extensive libraries; they become laborious and expensive tasks. Machine learning-guided methods are a cost-effective and less time-consuming strategy than acquiring data through in vitro and in vivo experiments. This strategy limits the number of candidates from large peptide libraries by predicting and ranking their biological activities from sequences, so-called Quantitative Structure–Activity/Property Relationship (QSA/PR) studies. Successful QSA/PR applications include the discovery of novel antimicrobial peptides^10–13^ or epitopes^14^ and, the design of anticancer peptides^15–17^. Besides to the predictive power of QSA/PR methods, it is essential to consider their limitations, which lie into those of supervised learning. Supervised learning regroups machine learning algorithms that require annotated training data. For instance, creating a classification model to predict the biological activity of any peptide sequence needs training on a vast number of sequences (or derived features thereof) that are labelled with their proper class of biological activity. The model has a limited space of reliable predictability known as the domain of applicability.

One of the problems associated with peptide-based drugs is their hemotoxic or hemolytic profiles. For instance, most antimicrobial peptides (AMPs) present in preclinical/clinical applications are applied topically, in part due to their hemolytic activity. Hemolysis is the disruption of erythrocyte membranes decreasing the life span of red blood cells and causing the release of haemoglobin. Antimicrobial peptides display direct antibacterial activities without or limited bacterial resistance offering promising avenues against antibiotic-resistant infections^3^. Identifying hemolytic AMPs and predicting their hemolytic activity is therefore critical to their applications as non-toxic and safe treatments against bacterial infections. In 2016–2017, two predictive online services for hemolytic activity emerged; HemoPred^18^ and HemoPI^19^. Both services provided predictions derived from sequence-based properties (PCP), AAindex^20^, amino acid composition (AAC) and sequence motifs (2-4mers) across publicly available HemoPI-1, HemoPI-2 and HemoPI-3 datasets. HemoPI-1 dataset helps to identify hemolytic peptides while HemoPI-2 and HemoPI-3 datasets serve for predicting high or low hemolytic potency. In April 2020, Hasan and co-workers published a third predictive online service named HLPpred-Fuse^21^ that simultaneously identified hemolytic peptides HLPs (using HemoPI-1 dataset) and predicted their high or low hemolytic activity (using HemoPI-3 dataset). The team has explored 54 features including AAC and PCP, across six binary classifiers. In July 2020, Timmons and Hewage^22^ reported a fourth online predictor for hemolytic activity, named HAPPENN, based on artificial neural network. The authors compiled a dataset of 3738 peptide sequences from which they derived several features including physicochemical descriptors. They compared their algorithms to HemoPI and HemoPred using HemoPI-2 and HemoPI-3 datasets resulting in higher performance metrics. All four online services—HemoPred^18^, HemoPI^19^, HLPpred-Fuse^21^, HAPPENN^22^—provide solid predictions of hemolytic peptides and their potency. None has however defined the domains of applicability of their models, which could extrapolate its predictive power after submitting novel sequences on its online platform.

This study expands into hemolytic QSA/PR models for the development of non-hemolytic peptides and safe AMP-based treatments. Considering the practicality, cost-effectiveness and time reduction of processing such models, we developed our models to identify (non-)hemolytic peptides and their potency from HemoPI datasets. We predicted the hemolytic nature and activity of 3081 AMPs (APD) and a dataset of 317 known hemolytic AMPs (HAMP) for external validation. We designed de novo 5000 random peptide sequences (RPS) enriching the number of non-hemolytic entities. We compared fourteen algorithms for binary classification including decision tree (CART), random forest (RF), gradient boosting (GBC), adaptive boosting (AB), logistic regression (LOGREG), support-vector machine (SVM) and K-nearest neighbours (KNN) classifiers, reported among aforementioned hemolytic predictors. We also assessed nine outlier detection (OD) methods to define the applicability domains of our models. Our study is the first application of multivariate OD methods to peptide-based QSA/PR modelling. To encourage dissemination and further implementation of OD methods into the existing predictive services, our pipeline (including generated data, exploratory analyses, predictive models), written in Python 3.6, are publicly available. Combining robust predictive models and OD methods guided our discovery of 34 non-hemolytic AMPs of natural origin, de novo design of 507 novel peptides and set guidelines for non-hemolytic peptide design.

## Methods

**Figure 1:**
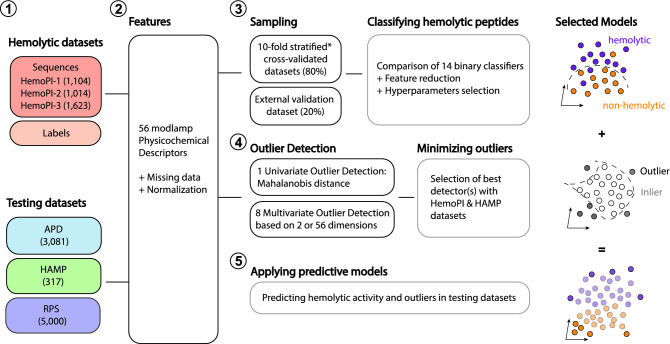
This workflow is showing different steps taken to discover non-hemolytic peptides from the Antimicrobial Peptide Database. From left to right, (1) collection of 10,000+ peptide sequences in 6 different datasets, (2) calculation of 56 physicochemical descriptors for each sequence, (3) development and selection of binary classifiers to predict the hemolytic activity using HemoPI datasets, (4) estimation of the applicability domain of selected models using univariate and multivariate outlier detectors and (5) application of optimised models to antimicrobial peptides datasets APD, HAMP and RPS.

Figure 1 summarizes the general workflow to discover non-hemolytic peptides from the Antimicrobial Peptide Database (APD) and design novel non-hemolytic peptides. In the first step, we assembled 6 different datasets comprising three publicly available datasets HemoPI-1, HemoPI-2 and HemoPI-3 for training as well as 3081 natural antimicrobial peptides from APD for testing and, 317 known hemolytic antimicrobial peptides (HAMP) for validation. In the second step, for each peptide sequence, we calculated 56 physicochemical properties (List S1). All datasets were cleaned up from missing and duplicated information, and they were normalized accordingly. In the third step, we developed models from 14 machine learning algorithms for binary classification in order to predict hemolytic activity from sequence-based physicochemical descriptors. We evaluated univariate and multivariate outlier detection methods to define the applicability domain of these models. Optimized models and outlier detectors were applied to the 3 testing datasets; 3081 natural antimicrobial peptides (APD dataset), 317 known hemolytic antimicrobial peptides (HAMP dataset) and 5000 de novo generated peptides. For the latter, we used a random sequence generator that required the amino acid frequencies and the range of sequence lengths of 2808 AMPs from APD (excluding 273 peptides with high outlier scores) to produce 5000 random peptide sequences (RPS). All computational studies were developed in Jupyter notebooks using various Python modules for scraping datasets, calculating sequence-based properties and developing exploratory data analysis and machine learning algorithms. Scripts are available at https://github.com/plissonf/ML-guided-discovery-and-design-of-non-hemolytic-peptides.

### Datasets

#### HemoPI datasets

HemoPI-1, HemoPI-2 and HemoPI-3 datasets consist of experimentally validated hemolytic peptides from Hemolytik database^23^ or extracted from Swiss Prot^24^ or Database of Antimicrobial Activity and Structure of Peptides (DBAASP v.2)^25^. Sequences were originally published by Chaudhary et al*.*^19^, and they are freely available for downloaded at https://webs.iiitd.edu.in/raghava/hemopi/datasets.php. HemoPI-1 contains 552 hemolytic peptides (Hemolytik) and 552 non-hemolytic peptides (Swiss Prot), HemoPI-2 contains 552 peptides with high hemolytic efficiency and 462 non-hemolytic peptides (Hemolytik), HemoPI-3 contains 885 peptides with high hemolytic efficiency and 738 low/non-hemolytic peptides (Hemolytik & DBAASP). All HemoPI datasets were split into two datasets—1 main/model dataset (80%) used for model building and 1 smaller dataset (20%) used as external validation.

#### APD dataset

3081 antimicrobial peptide sequences and related information were scraped from The Antimicrobial Peptide Database^26^ website https://aps.unmc.edu/AP/main.php (November 2019). Of note, 132 peptides from APD matched in HemoPI-1, 229 peptides in HemoPI-2 and 476 peptides in HemoPI-3.

#### Hemolytic AMPs (HAMP)

All 317 hemolytic peptides were extracted from our APD dataset using “hemolytic” activity as filter (November 2019). Of note, 63 peptides from HAMP matched in HemoPI-1, 67 peptides in HemoPI-2 and 147 peptides in HemoPI-3.

#### Random peptide sequences (RPS)

All 5000 sequences were generated using modlamp^27^ “sequences.Random” module that requires the amino acid composition [A: 0.08, C: 0.06, D: 0.02, E: 0.02, F: 0.05, G: 0.11, H: 0.02, I: 0.07, K: 0.11, L: 0.11, M: 0.01, N: 0.03, P: 0.04, Q: 0.02, R: 0.05, S: 0.06, T: 0.04, V: 0.06, W: 0.02, Y: 0.02] and the range of sequence lengths (min: 21–max: 38) from APD.

### Physicochemical properties

Using Python-based package modlamp^27^, we calculated 56 physicochemical properties (47 peptide and 9 global descriptors) from all primary sequences. For the definitions of all 56 properties, see Supporting Information List S1. All properties were assembled as columns into datasets, and peptide sequences are rows.

### Pre-processing datasets

All sequences with duplicated information and/or missing values were removed. All HemoPI model and validation datasets were normalised as *X*_*HemoPI*_ values using Eq. (1). Testing datasets APD, HAMP and RPS datasets were normalised as *X*_*test*|*HemoPI*_ values relative to the model dataset in use for predictions with Eq. (2). Values *x*, *x*_*min*_ and *x*_*max*_ belong to the model dataset such as HemoPI-1 and, *x*_APD_ belongs to the testing set, e.g. APD.

1 $$X_{HemoPI - 1} = \frac{{\left( {x - x_{min} } \right)}}{{\left( {x_{max} - x_{min} } \right)}}$$

2 $$X_{APD| HemoPI - 1} = \frac{{\left( {x_{APD} - x_{min} } \right)}}{{\left( {x_{max} - x_{min} } \right)}}$$

### Machine learning algorithms

In this study, we evaluated a list of 14 binary classification algorithms to predict haemolytic activity from sequence-based physicochemical properties that includes Logistic Regression LOGREG^28^, K-nearest neighbour KNN^29^, Linear and Quadratic Discriminant Analysis LDA/QDA^30^, Support Vector Classifier SVC (with the 4 kernels linear, radial basis function, polynomial, sigmoid)^31^, Decision Tree CART^32^, Random Forest Classifier RFC^33^, Gradient Boosting Classifier GBC^34^, Adaptive Boosting Classifier ABC^35^, Extreme Gradient Boosting Classifier XGBC^36^. All algorithms were computed using Python package Scikit-learn 0.23.1^37^. Gradient Boosting classifiers ranked model features in order of importance as shown in Fig. S1a–c.

### Performance metrics

To evaluate the performances of all classifiers, we used the following assessment measures: accuracy (Acc.) (Eq. (3)), precision (Prec.) or positive predictive value (PPV) (Eq. (4)), Matthews correlation coefficient (MCC) (Eq. (5)), Cohen’s Kappa statistic (CK or κ) (Eq. (6)) and area under the curve Receiver Operating Characteristic (AUC-ROC) value (see Tables 1, 2, 3 and Tables S1a–S5).

3 $$Acc. = \frac{TP + TN}{{TP + TN + FP + TN}}$$

4 $$Prec. \; or\; PPV = \frac{TP}{{TP + FP }}$$

5 $$MCC = \frac{TP \times TN - FP \times FN}{{\sqrt {\left( {TP + FP} \right) \times \left( {TP + FN} \right) \times \left( {TN + FP} \right) \times \left( {TN + FN} \right)} }}$$

6 $$CK or = \frac{{P_{O} - P_{E} }}{{1 - P_{E} }}$$

where true positive (TP) is the number of true hemolytic peptides that are predicted correctly; true negative (TN) is the number of true non-hemolytic peptides that are predicted correctly; false positive (FP) is the number of true hemolytic peptides that are predicted to be non-hemolytic; true negative (TN) is the number of true non-hemolytic peptides that are predicted to be hemolytic; P_O_ is the relative observed agreement among raters and P_E_ is the hypothetical probability of chance agreement.

**Table 1:** Performance of 14 binary classifiers for predicting hemolytic activity using 3 model datasets (HemoPI-1, HemoPI-2, HemoPI-3) and 56 sequence-based physicochemical descriptors.

Classifiers	Mean accuracy (%)					
	HemoPI-1 dataset		HemoPI-2 dataset		HemoPI-3 dataset	
	Model	Validation	Model	Validation	Model	Validation
LOGREG	92.6	89.6	68.0	65.8	69.7	70.8
KNN	93.2	86.8	71.6	63.4	72.8	65.8
CART	90.4	83.7	69.6	62.4	68.9	64.0
RFC	93.8	87.3	69.0	63.9	72.4	66.8
**GBC**	**94.0**	**90.4**	**76.7**	**72.3**	**75.8**	**72.9**
ABC	93.8	91.4	71.3	73.8	72.3	71.4
**LDA**	**94.2**	**90.5**	70.0	59.9	70.8	70.8
QDA	91.5	85.5	66.4	60.9	65.9	59.4
NB	85.9	85.5	62.3	63.4	64.4	65.9
SVC-LIN	88.1	85.0	61.9	59.9	65.4	66.5
SVC-RBF	88.7	85.5	63.7	62.4	66.3	66.2
SVC-POLY	71.5	60.0	54.4	54.5	54.6	54.5
SVC-SIG	88.0	81.8	59.6	54.5	63.4	63.4
**XGBC**	**94.8**	**92.4**	76.1	69.3	**74.7**	**73.2**

All performance metrics (Accuracy, Precision, Matthews correlation coefficient, Cohen’s kappa statistic and Receiver operating characteristic area under curve) are available in Supporting Information.

The best binary classifiers and their respective performances are depicted in bold.

**Table 2:** Three top performing binary classifiers for HemoPI-1, HemoPI-2, HemoPI-3 datasets after optimizing classifier hyperparameters and number N of descriptors.

Classifiers		Hyperparameters	Feature reduction	N	Acc. (%)	Prec. (%)	MCC statistic	CK statistic	AUC ROC
**HemoPI-1 model and validation datasets**									
1.1	LDA	'solver': 'svd', 'tol': 0.0001	RFECV	18	95.1	92.6	0.903	0.903	0.951
					94.6	92.5	0.891	0.891	0.946
1.2	GBC	'max_depth': 4, 'max_features': 'sqrt', 'min_samples_leaf': 10, 'n_estimators': 240	MC (0.75)	26	96.5	95.0	0.930	0.930	0.965
					92.7	89.6	0.855	0.855	0.927
1.3	GBC	'max_depth': 4, 'max_features': 'sqrt', 'min_samples_leaf': 10, 'n_estimators': 208	None	56	96.0	94.6	0.921	0.921	0.960
					92.3	89.2	0.846	0.846	0.923
**HemoPI-2 model and validation datasets**									
2.1	GBC	'max_depth': 4, 'max_features': 'sqrt', 'min_samples_leaf': 2, 'n_estimators': 112	None	56	77.7	74.0	0.549	0.549	0.774
					74.3	70.4	0.479	0.476	0.736
2.2	GBC	Default	RFECV	15	77.8	74.2	0.552	0.552	0.775
					73.2	69.8	0.459	0.482	0.728
2.3	GBC	Default	None	56	76.7	72.9	0.529	0.528	0.763
					72.3	68.9	0.439	0.437	0.717
**HemoPI-3 model and validation datasets**									
3.1	GBC	'max_depth': 18, 'max_features': 'log2', 'min_samples_leaf': 10, 'n_estimators':192	None	56	80.0	76.4	0.597	0.597	0.796
					71.7	68.3	0.427	0.425	0.711
3.2	GBC	'max_depth': 12, 'max_features': 'sqrt', 'min_samples_leaf': 8, 'n_estimators': 160	RFECV	40	78.2	74.4	0.559	0.558	0.777
					74.5	70.8	0.483	0.482	0.740
3.3	GBC	'max_depth': 20, 'max_features': 'log2', 'min_samples_leaf': 8, 'n_estimators': 96	MC (0.75)	28	78.0	74.4	0.556	0.556	0.777
					72.6	69.0	0.445	0.443	0.719

Optimal number N of descriptors were determined using multicollinearity, RFECV: tenfold cross-validated recursive feature extraction or BE: backward elimination.

**Table 3:** Top performing Extreme Gradient Boosting classifiers (XGBoost) for HemoPI-1, HemoPI-2, HemoPI-3 datasets before and after optimizing classifier hyperparameters and number N of descriptors.

Hyperparameters	Feature reduction	N	Acc. (%)	Prec. (%)	MCC statistic	CK statistic	AUC ROC
**HemoPI-1 model and validation datasets**							
Default	MC (0.75)	26	95.7	93.7	0.914	0.914	0.957
			92.3	88.5	0.846	0.846	0.923
**HemoPI-2 model and validation datasets**							
'colsample_bytree': 0.8, 'eta': 0.1, 'max_depth': 14, 'min_child_weight': 1, 'subsample': 0.7, 'tree_method': 'hist', 'objective':'binary:logistic'	RFECV	34	79.1	75.3	0.577	0.577	0.787
			70.3	67.2	0.398	0.397	0.697
**HemoPI-3 model and validation datasets**							
'colsample_bytree': 0.8, 'eta': 0.2, 'max_depth': 14, 'min_child_weight': 0.2, 'subsample': 0.8, 'tree_method': 'approx', 'objective':'binary:logistic'	MC (0.75)	28	78.7	74.9	0.569	0.568	0.783
			72.6	69.1	0.445	0.444	0.720

Optimal number N of descriptors were determined using MC: multicollinearity and RFECV: recursive feature extraction with tenfold cross-validation.

### Class and class probabilities

Each peptide sequence was output a class (e.g. 0: non-haemolytic and 1: haemolytic peptide) and a probability P to belong to that same class that varies between 0.00 and 1.00 (e.g. P(1) = 0.67). For each sequence, the sum of class probabilities P(0), P(1) is equal to 1.

### Cross-validation techniques

Performances of our classification models were evaluated using two cross-validation techniques based on the class balance in HemoPI datasets. We applied tenfold cross-validation with the balanced HemoPI-1 dataset and stratified tenfold cross-validation with the imbalanced datasets; HemoPI-2 and HemoPI-3. In tenfold cross-validation, sequences are randomly divided into 10 subsets (folds); 9 sets train the models and, the remaining set is the internal test set. Stratified tenfold cross-validation is a variant of tenfold cross-validation where the folds are stratified, which means they preserved the percentage of sequences for each class.

### Feature elimination

We reduced the number of variables/features (i.e. physicochemical properties) associated with each class to keep only the most informative and non-redundant ones. We applied three feature elimination/extraction approaches so-called Recursive Feature Elimination with tenfold Cross-Validation RFECV^38^, Backward Extraction BE^39^ and Multicollinearity MC^40^. RFECV selects variables into smaller and smaller sets before tuning the final number of variables using cross-validation. BE or stepwise regression tests and deletes variables that do not fit with the class column in a stepwise manner. MC excludes all highly correlated variables (based on a correlation coefficient cut-off) to only keep non-redundant properties.

### Hyperparameter tuning using GridSearchCV

For each classifier, we chose a limited number of specific hyperparameters (e.g. SVC: *C* and *gamma*). All chosen hyperparameters can be seen in Tables S4a–c and S5b. For each hyperparameter, we defined a range of values or several labels according to the *Parameters* instructions given in Scikit-learn 0.23.1^37^. To tune hyperparameters, we run a grid search with cross-validation (GridSearchCV) that evaluate all possible combinations of hyperparameters’ values or labels and select the optimal combination based on model accuracy score.

### Unsupervised outlier detection

We detected novelties/outliers from our datasets by applying univariate or multivariate outlier detection methods. For univariate detection, we detected the outliers using Mahalanobis distance (MD)^41^. MD is an effective distance metric that measures the distance between a point and a distribution in multivariate space, i.e. 56 sequence-based physicochemical properties. The formula to compute Mahalanobis distance is as follows (Eq. (7)):

7 $$D^{2} = \left( {x - m} \right)^{T} \times C^{ - 1} \times \left( {x - m} \right)$$

where *D*^2^ is the squared Mahalanobis distance, *x* is the input vector (row in a dataset), *m* is the vector of mean values of independent variables (mean of each column) and *C*^−1^ is the inverse covariance matrix of independent variables.

Alternatively, we detected multivariate outliers directly from high dimensional space using proximity-based methods (LOF: local outlier factor^42^, CBLOF: clustering-based local outlier factor^43^, HBOS: histogram-based outlier score^44^, (Average) KNN: (Average) K-nearest neighbours^45^), outlier ensembles (IF: isolation forest^46^, FB: feature bagging^47^) and the probabilistic method angle-based outlier detection or ABOD^48^. All algorithms were computed using PyOD, a python toolbox for scalable outlier detection (https://pyod.readthedocs.io/)^49^. In HemoPI and HAMP datasets, sequences that deviate from the overall (uni- or multivariate) distribution are experimentally validated as (non-)haemolytic peptides; they are referred to as *novelties*. In testing datasets APD and RPS, these sequences are not labelled; they are true *outliers*.

### Outlier scores

Each peptide sequence acquires an outlier score (OS) that differs according to the multivariate outlier detectors. The higher this score is, the more outlying the peptide is considered. Outliers tend to have higher scores. For example, in Fig. 4, outlier scores vary between 0.0 and 1.0, and outliers are labelled as “dark-coloured” data points (i.e. peptide) with scores higher than 0.99 (91^st^ percentile). Percentile outlier scores (POS) are the percentage of outlier scores in its frequency distribution. In Fig. 5, percentile outliers scores are shown at 0.25 (1^st^ quartile/25^th^ percentile, OS = 0.54), 0.50 (2^nd^ quartile/50^th^ percentile, 0.69), 0.75 (3^rd^ quartile/75^th^ percentile, 0.89) and 0.91 (outlier threshold, 0.99) for a distribution of outlier scores varying between 0.0 and 2.0.

### Dimensionality reduction

In addition to Mahalanobis distance, we explored 17 dimensionality reduction techniques to visualize the distribution of peptide libraries from their 56 physicochemical dimensions, or peptide property space, into bi-dimensional representations. We evaluated these techniques with HemoPI-1 model dataset using R_NX_(K) quality curves^50^ (Fig. S2) using the dimRed R package^51^. Among the different techniques, we selected t-distributed stochastic neighbour embedding (t-SNE) to display HemoPI-1, AMP and HAMP datasets. T-distributed stochastic neighbour embedding is a visualization technique well suited for high-dimensional data that display observations onto lower dimensions guided by a non-convex objective function^52^.

### Amino acid composition

Amino acid frequencies were calculated for each peptide sequence, and they were averaged for each dataset (e.g. HemoPI-1, Fig. 6).

### Statistical tests

We measured normality of dataset distributions (i.e. physicochemical properties) for both groups (inliers and outliers) using the Lilliefors test before evaluating which dataset(s) had the same distribution in both groups. We determined the variance with either F-test for a normally distributed dataset (ND) or Fligner-Killen test for an abnormally distributed dataset (AD). We compared the means of physicochemical properties between inliers and outliers by applying the three respective statistical tests; (1) Welch’s t-test to NDs with different variances, (2) Wilcoxon test (also known as Wilcoxon rank-sum) to ADs with the same variance and (3) Kolmogorov–Smirnov test to ADs with different variances, using a significance level α of 0.001 (or 0.1%). We controlled the false discovery rate with Benjamini and Hochberg method using the same value α. All tests were performed using statistical software R version 3.6.3/R studio version 1.2.5033^53,54^. The statistical pipeline is visible in Fig. S3.

### Differential cumulative frequencies

We measured the enrichment of 5000 generated inliers sequences in charged, small and bulky amino acids using differential cumulative frequencies of selected amino acids as follows in Fig. 7c (Eq. (8)) and Fig. 7d (Eq. (9)). Similar amino acid analysis was conducted for the 3081 peptides from APD dataset as shown in Figure S5.

8 $${\Sigma } f_{K, R, H} - {\Sigma } f_{D, E}$$

9 $${\Sigma } f_{G, C, A, P, S} - {\Sigma } f_{L,I, F, Y, W}$$

## Results

### Predicting the hemolytic nature and activity of antimicrobial peptides

The predictive power of machine learning models depends on several factors; the input datasets, the quality of independent variables/features and the type of algorithms (i.e., classifiers, regressors). In order to compare our models with the performances of online services HemoPred^55^, HemoPI^19^ and HLPpred-Fuse^21^ to predict the hemolytic nature and activity of peptides, we used the 3 publicly available HemoPI datasets. All peptides were embedded into 56 sequence-based physicochemical properties that were calculated using a Python-based software modlAMP^27^. We selected an extensive list of 14 algorithms commonly used for binary classification including aforementioned 7 algorithms (CART, RF, GBC, ABC, LOGREG, SVM and KNN), which have been evaluated to build HemoPred^55^, HemoPI^19^ and HLPpred-Fuse^21^ classifiers. Their performances are summarized in Table 1 (and extended Supporting Information Tables S1a–c and S5a–b) for all HemoPI model and validation sets.

Overall, the hemolytic nature of peptides was predicted with higher accuracies than their hemolytic activities. HemoPI-1-based models led with model accuracies at 94–95% and validation accuracies at 90–92% while HemoPI-2/3-based models capped at 75–77% and 70–73%, respectively, for the same algorithms. Gradient boosting machines (GBC) outperformed the first 13 classifiers with model accuracies of 94.0, 76.7 and 75.8% across respective HemoPI datasets. Tree-based adaptive boosting (ABC) and linear discriminant analysis (LDA) classifiers demonstrated comparable performances with HemoPI-1 model accuracies at 93.8–94.2% and validation accuracies at 90.5–91.4%. Interestingly, random forest (RFC) and support vector (SVC) classifiers that were recommended by other hemolytic predictors, did not perform as well with our modlAMP features. For example, support vector classifiers with linear, radial basis function and sigmoid kernels predicted the hemolytic nature of peptides (HemoPI-1 dataset) with 88.0–88.7% model accuracy. In contrast, Chaudhary and co-workers reported SVM with five-fold cross-validated model accuracy at 95.3%^19^. Our basic random forest classifier exhibited Matthews correlation coefficients (MCC) of 0.88 (see Table S1a) while Win and co-workers reported RFC with MCC of 0.92^55^. Given the promising performances of boosting classifiers GBC and ABC across all HemoPI datasets, we evaluated extreme gradient boosting classification (XGBC) by implementing XGBoost with default hyperparameters. XGBC models, based on HemoPI-1 and HemoPI-3 datasets, improved by 0.3–2.0 points to their basic gradient boosting classifiers, as outlined in Table 1 and Supporting Information Table S5a.

Next, we selected the 6 binary classifiers LOGREG, RFC, GBC, LDA, SVC-RBF (kernel with radial basis function) and XGBC for further optimization. First, we attempted to improve our models by removing redundant and/or non-informative variables using 3 feature elimination techniques—multicollinearity (MC), recursive feature elimination with cross-validation (RFECV) and backward elimination (BE). The mean accuracies are summarized for the first 5 classifiers in Table S2 and for extreme gradient boosting, see Table S5a. To reduce the number of variables with multicollinearity, we evaluated correlation coefficient cut-offs ranging from 0.75 to 0.95, and we compared the performances (mean accuracies) implementing gradient boosting classification across HemoPI-1, 2 and 3. We identified the optimal cut-off of 0.75, common to all HemoPI datasets, based on the best overall model and validation accuracies, as indicated in Table S3. Of the three feature elimination techniques tested, both MC and RFECV led to higher model and validation accuracies by 1.0 to 4.0 points (Table S2). For instance, our gradient boosting classifier with all 56 variables (physicochemical descriptors) predicted the hemolytic nature of peptides (HemoPI-1) with 94.0% and 90.4%, model and validation accuracies, respectively. With a reduced HemoPI-1 dataset of 26 variables by multicollinearity, the performances of GBC reached 95.4% and 91.8% accuracies. Likewise, extreme gradient boosting classifier (XGBC) exhibited 95.7% and 92.3% accuracies (Table S5a).

Linear discriminant analysis (LDA) performed similarly to default GBC with 94.2% and 90.5% accuracies on the same 56-dimensional dataset. After applying cross-validated recursive feature elimination, LDA performances peaked at 95.1 and 94.5% accuracies, outperforming XGBC. With regards to reduced HemoPI-2 and HemoPI-3 datasets by multicollinearity, GBCs displayed higher model accuracies but lower validation accuracies. Gradient boosting classifier improved by 1.0 point for both model and validation accuracies at 77.8 and 73.3%, respectively, using 15-dimensional HemoPI-2 dataset reduced by cross-validated recursive feature elimination (Table S2). Unlike gradient boosting classifiers, XGBC models display similar performances to models with the complete HemoPI-2 and HemoPI-3 datasets (Table S5a). Second, we aimed at improving our models by fine-tuning their specific hyperparameters using GridSearchCV across all HemoPI datasets. We compared these models to the ones with HemoPI datasets that were reduced either by multicollinearity and cross-validated recursive feature elimination. All results are summarized in Supporting Information Tables S4a–c and S5b. All models were compared to one another based on the overall performances for both model and validation datasets. In Table 2, we gathered the three best performing binary classifiers for each HemoPI dataset, with their respective tuned hyperparameters, the final number of variables and performance metrics. Optimized LDA and GBC models predicted the hemolytic nature of peptides at 95.1–96.5% model accuracies and 92.3–94.6% validation accuracies. For HemoPI-2 and HemoPI-3 datasets, our best performing models are essentially based on gradient boosting algorithm. With HemoPI-2 dataset, GBC models peaked at 76.7–77.8% and 72.3–74.3% accuracies with the complete set of variables and/or default hyperparameters. With HemoPI-3 dataset, GBC models could predict the high or low/non- hemotoxicity of peptides with accuracies at 78.0–80.0% and 71.7–74.5%. Here, we noted a progressive loss in performances as the number of variables diminished. In Table 3, we selected the three best performing extreme gradient boosting classifiers, one per HemoPI dataset. Overall, the three XGBC models achieved similar or inferior performances compared to selected GBC models presented in Table 2.

Considering that a classifier accuracy could be sensitive to class imbalance (i.e. HemoPI-2 and -3 datasets), we compared and selected our binary classifiers with the following additional metrics; precision (%), the area under Receiver Operating Characteristic curve (AUC ROC), Matthews correlation coefficient (MCC) and Cohen’s κ score (CK) as depicted in Tables 2 and 3. Most precision and AUC ROC values were in the same order of magnitude as reported model and validation accuracies. For binary classification, MCC is the computed correlation coefficient, and CK is the degree of agreement between true classes and predicted classes. We observed that these values were quasi-identical across all datasets and models. For HemoPI-2 and -3 datasets, MCC and CK values ranged between 0.40 and 0.60 that indicated strong positive relationships and moderate agreements between true and predicted values, respectively. For HemoPI-1, MCC and CK values were close to 0.90, suggesting nearly perfect agreement between observations and predictions. These results showed that our selected models, listed in Tables 2 and 3, were accurate and robust binary classifiers across the different datasets.

Except for model 1.1, all of our top binary classifiers grounded on the gradient boosting algorithm that has a built-in estimation of variable/feature importances (see Table 2). These importances were laid out for each dataset and their respective GBC models in Supporting Information Figure S1a–c. We observed that variables that contribute the most to the binary classification differed between two or three models using the same dataset. For example, both classifiers 1.2 and 1.3 that predicted the hemolytic nature of peptides using HemoPI-1 dataset, shared similar hyperparameters and performance metrics. Nevertheless, model 1.3 used the complete set of 56 variables, while model 1.2 utilized only 26 non-redundant variables. In results, the former model identified hemolytic peptides from differences in charge (net charge, charge_phys, charge_acid, isoelectric point), size (length, molecular weight) and polarity (polarity, ISAECI) at the amino acid and global levels. The latter distinguished hemolytic and non-hemolytic peptides using composite properties (Z5_1/5, Z3_1/2/3, Grantham), hydrophobicity (uH_Eisenberg, H_Eisenberg, H_GRAVY) and solubility (uS_AASI)—see Supporting Information Figure S1a. We discerned similar trends from high-dimensional models 2.1 and 2.3 compared to low-dimensional model 2.2 that predicted high hemolytic activity or none using HemoPI-2 dataset—see Supporting Information Figure S1b. Gradient Boosting classifiers that predicted high versus low hemolytic activity of peptides from HemoPI-3 dataset, displayed subtle differences in variable importances—see Supporting Information Figure S1c. In model 3.1, the most important variables were about size (molecular weight) as well as moments of shape (u_MSS_shape, uB_Bulkiness), refractivity (u_refractivity), polarity (u_polarity) and hydrophobicity (uH_Janin, uH_Eisenberg, etc.) at the amino acid scale. In model 3.2, peptides were classified based on their differences in polarity (polarity, ISAECI, modlabs_ABHPRK), hydrophobicity (uH_KyteDoolittle, uH_argos, etc.) and alpha-helical propensity (uF_Levitt). Finally, model 3.3 differentiated hemolytic peptides with high or low efficiency using composite properties (Z5_2, Z5_5, modlabs_ABHPRK, Grantham, etc.), hydrophobic scales (uH_Eisenberg, H_HoppWoods, uH_argos, H_GRAVY) and bulky amino acids (uB_Bulkiness, B_Bulkiness). All properties are described in the Supporting Information List S1.

### Defining the applicability domains of hemolytic models

**Figure 2:**
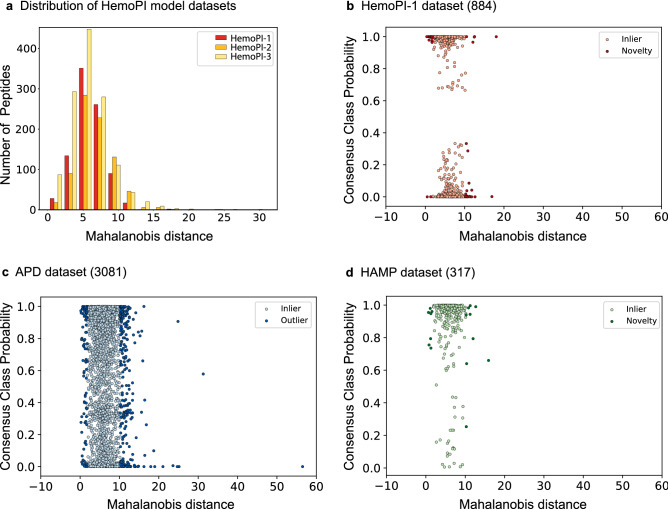
Distribution of Mahalanobis distances for HemoPI-1, HemoPI-2 and HemoPI-3 model datasets (**a**). Projections using Mahalanobis distances and consensus class probabilities from HemoPI-1 models—HemoPI-1 model dataset (**b**), APD dataset (**c**) and known hemolytic AMPs from APD dataset HAMP (**d**). Inliers (light colours) and novelties/outliers (dark colours) were classified based on empirical rule (99.7% normal distribution) applied to Mahalanobis distances.

The applicability domain (AD) characterizes a specific region in the underlying property space where the model predictions are considered reliable. That property space is a composite projection in N dimensions (N ≤ 56) that resulted from some or all physicochemical descriptors. The boundaries of AD lie into the diversity of peptide sequences and biological information. The most common way to delineate these boundaries uses unsupervised detection of outliers. An outlier is an observation (e.g. peptide) that deviates from an overall pattern in a dataset. There are two kinds of outliers, univariate and multivariate. Univariate outlier detection methods identify outliers as extreme values of a distribution in a single variable space such as standard Z-score. With 56 normalized variables per peptide, we instead evaluated our observations with different multivariate outlier detection methods. First, we used Mahalanobis distance (MD) that identifies outliers based on the empirical rule (99.7% normal distribution). MD reduced the multidimensional HemoPI datasets to a distance metric that reflected their general distributions onto a single scale, as depicted in Fig. 2a. Combined with consensus class probabilities (average values) resulting from models 1.1–1.3, we unveiled the distributions of 884 peptides from HemoPI-1 model dataset, 3081 antimicrobial peptides from APD and 317 known hemolytic antimicrobial peptides in HAMP (Fig. 2b–d). In Fig. 2b, we could see a clear separation between hemolytic and non-hemolytic peptides with their consensus class probabilities either above 0.6 or below 0.4. Some 47 observations from both classes positioned at extreme MD values (in dark red, fraction = 0.05 or 5%), they were called novelties. They represented a novel space, a sparsely populated space of known hemolytic or non-hemolytic peptides. With the APD dataset, we counted 337 outliers across the entire range of consensus class probabilities (in dark blue, 11%, Fig. 2c). In Fig. 2d, our models 1.1–1.3 have correctly predicted most known hemolytic antimicrobial peptides (HAMP) with consensus class probabilities above 0.5–0.6, where we identified 16 novelties (in dark green, 5%). We conducted similar MD-based outlier detections with HemoPI-2 and HemoPI-3 datasets. We also measured new MD values for APD and HAMP datasets, and we detected their respective new outliers within the property spaces of the model datasets, as summarised in Table S6.

**Figure 3:**
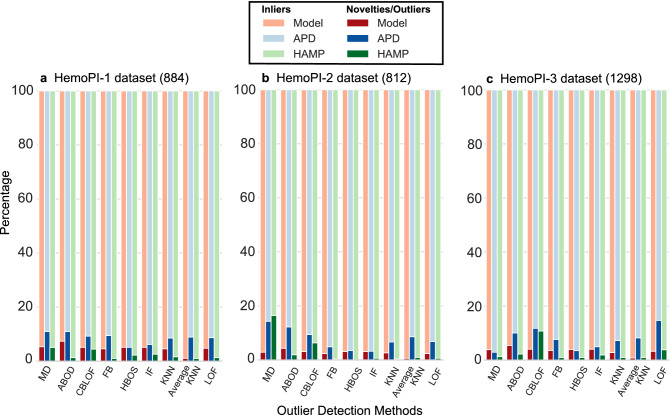
Percentages of inliers (dark colours) and novelties/outliers (light colours) applying univariate (MD: Mahalanobis distance) and multivariate outlier detection methods to 56-dimensional model datasets HemoPI-1 (**a**), HemoPI-2 (**b**), HemoPI-3 (**c**) (in reds), Antimicrobial Peptide Dataset (APD, 3081—in blues) and known hemolytic antimicrobial peptides in APD (HAMP, 317—in greens).

Transforming high-dimensional data (e.g. 56 physicochemical properties) into a single dimension (e.g. Mahalanobis distance) to detect outliers faces some challenges grouped under the umbrella term “curse of dimensionality”^56^. These challenges have motivated the development of alternative outlier detection methods. We benchmarked 8 of these multivariate methods; namely proximity-based methods (LOF: local outlier factor^42^, CBLOF: clustering-based local outlier factor^43^, HBOS: histogram-based outlier score^44^, (Average) KNN: (Average) K-nearest neighbours^45^), outlier ensembles (IF: isolation forest^46^, FB: feature bagging^47^) and the probabilistic method ABOD: angle-based outlier detection^48^. With these multivariate outlier methods, we had to inform with an outlier fraction for the 3 HemoPI datasets. In order to compare these methods with Mahalanobis distance, we chose outlier fractions reported in Table S6, i.e. 0.05 for HemoPI-1, 0.03 for HemoPI-2 and 0.04 for HemoPI-3 datasets. Our results to detect novelties/outliers across all HemoPI datasets (in reds) and in testing datasets, APD (in blues) and HAMP (in greens), are illustrated as percentages in Fig. 3, including MD-based results. Numeric values are gathered in Table S7. Overall, we observed that the number of novelties and outliers, depicted in darker colours, represented less than 10–20% of their respective datasets. The percentage of peptides with extreme MD values was often higher among antimicrobial peptides than other datasets of (non-)hemolytic peptides (HemoPIs, HAMP), in part due to the size and diversity of peptide sequences in APD. In the absence of labelled or predetermined outliers (unsupervised detection), we selected the best outlier detector as the method that encompasses the maximum number of observations (inliers) from each HemoPI model dataset. In other words, the best method must identify the boundaries with the lowest numbers of novelties (novel space) in a model property space.

**Figure 4:**
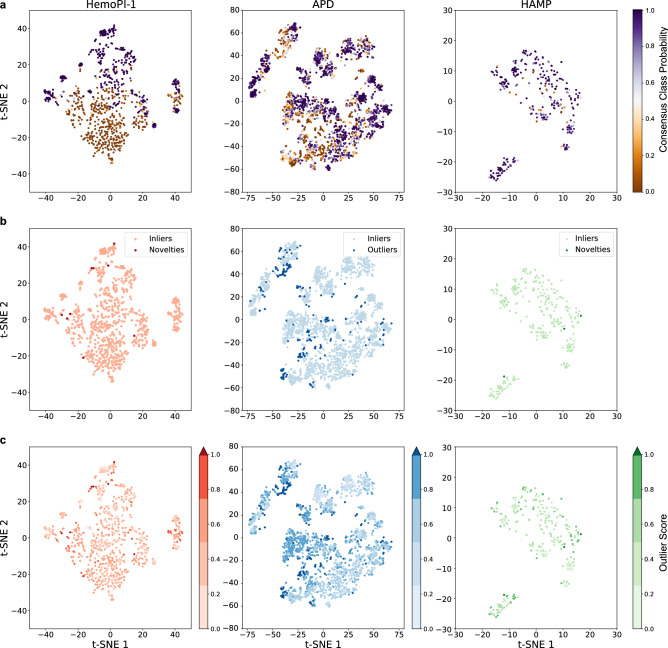
t-SNE projections showing the distributions of HemoPI-1 model, APD and HAMP datasets with the following labels; (**a**) their respective consensus class probabilities, (**b**) their classifications as inliers, outliers or novelties and, (**c**) their outlier scores predictions.

Based on this assumption, we picked Average K-Nearest Neighbour (Average KNN) as the best multivariate outlier detection approach for all HemoPI datasets. Average KNN scored the lowest number of novelties simultaneously across the property spaces of 3 HemoPI datasets and the corresponding projections of HAMP dataset. For instances, Average KNN identified 14 peptides in HemoPI-1 (1.6%) and 3 peptides in HAMP (~ 1%) as novelties and, 273 AMPs (8.9%) from APD as outliers as shown in Fig. 3a. With HemoPI-2 dataset, the number of novelties lowered to 5 novelties (0.6%) in the model dataset and remained at 3 in HAMP and, we counted 264 APD outliers (8.6%)—Fig. 3b. Finally, Average KNN resulted in 10 novelties (1.2%) in HemoPI-3 dataset, 3 novelties in HAMP and 253 APD outliers (8.2%)—Fig. 3c. Coincidentally, only with Average KNN, we noted that the number of novelties and outliers decreased as the number of peptides in model datasets (884 in HemoPI-1 versus 1256 in HemoPI-3) increased. In an attempt to visualize more clearly the distributions of novelties, outliers and inliers across datasets, we reduced HemoPI-1, APD and HAMP from 56 to 2 dimensions applying the non-linear dimensionality reduction technique known as t-distributed stochastic neighbour embedding (t-SNE). Compared to 16 other dimensionality reduction techniques, t-SNE scored the highest AUC value of 0.51, suggesting this embedding to be the most appropriate to display HemoPI-1 dataset (Fig. S2). We presented these distributions into bi-dimensional scatterplots with different (coloured) labels (Fig. 4). For the sake of clarity and visual attraction, we intentionally kept different scales on t-SNE1/t-SNE2 axes for each dataset.

First, we reported the distributions of HemoPI-1, APD and HAMP datasets (from left to right) according to their consensus class probabilities as shown in Fig. 4a. On the top left corner of that figure, we could almost distinguish between the two classes of HemoPI-1; i.e. hemolytic peptides (in shades of purple) and non-hemolytic peptides (in shades of orange). In contrast, most APD antimicrobial peptides gathered into several clusters of low or high consensus class probabilities overlapped. As for Fig. 2d, the dataset of known hemolytic peptides (HAMP) mostly distributed in high consensus class probabilities. In Fig. 4b, we displayed the same datasets, where novelties and outliers outlined in dark colours and, inliers in light colours. We recognized the 14 HemoPI-1 novelties (in dark red), 273 APD outliers (in dark blue) and 3 HAMP novelties (in dark green) as previously identified in Fig. 3a. Figure 4c pictured the three datasets, not as discrete classes inliers/outliers/novelties but, along a continuous gradient, the outlier score. The darker is the colour; the higher is the outlier score. Regardless of the dataset, observations (i.e. peptides) with outlier scores higher than 0.99 (percentile outlier score > 0.91) were classified as either outliers or novelties.

### Discovering non-hemolytic AMPs within the APD universe

**Figure 5:**
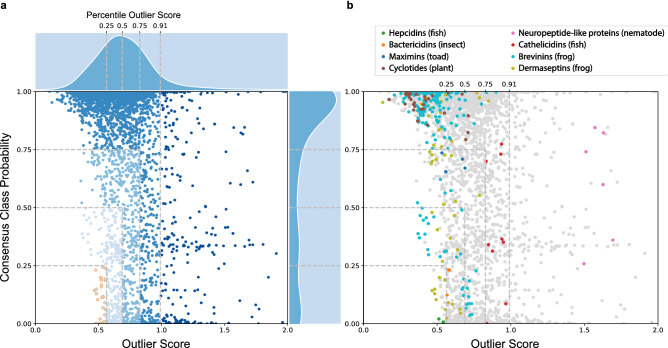
(**a**) Scatterplot showing the distribution of 3081 AMPs according to outlier scores and hemolytic (HemoPI-1) consensus class probabilities. Changes in the outlier scores and probabilities are illustrated with different shades of blues. Selected 34 non-hemolytic AMPs (golden circles) with lowest outlier scores and hemolytic predictions. Darkest data points are 273 AMPs detected as outliers according to HemoPI-1 Average KNN with percentile outlier scores > 0.91. (**b**) Colour-coded antimicrobial peptide families (e.g. dermaseptins) from different source organisms.

Among 3081 antimicrobial peptides from the APD dataset (Fig. 4, centre), we identified 317 peptides with hemolytic activity that we gathered under the acronym HAMP (Fig. 4, right). These hemolytic antimicrobial peptides represented only 10% of the APD dataset. Considering that hemolytic assays are not conducted routinely in laboratories once an antimicrobial peptide is isolated or synthesized, our QSA/PR models become essential to predict its hemolytic nature or activity. We applied our 3 best HemoPI-1 models (1.1–1.3, Table 2) to both APD and HAMP datasets and reported their consensus class probabilities to be hemolytic peptides, as we first presented in Fig. 2c–d as well as in Fig. 5. In Fig. 2d, 288 HAMPs (90.8%) had their consensus class probabilities above 0.6 (or 272–85.8% above 0.75), which validated the correct predictions of our models. In contrast, we predicted a handful of frog and insect AMP families, e.g. dermaseptins, ocellatins, odorranains, phylloseptins, bactericidins as non-hemolytic peptides (with consensus class probabilities below 0.5). Figures 2c and 5 showed that roughly two-thirds of the 3081 APD peptides (2084–67.6%) were predicted to be hemolytic peptides, which supports the common belief that natural AMPs are considered toxic due to their hemolytic activity. In Fig. 5, we showed the distribution of the APD dataset according to their consensus class probabilities and outlier scores. In Fig. 5a, this distribution is divided into five quadrants with specific cut-offs and five different colour schemes. We determined APD inliers using Average KNN with percentile outlier score lower than 0.91 (or outlier scores lower than 0.99). Starting in the bottom left corner, we identified 34 AMPs (golden circles) with the lowest class probabilities and percentile outlier scores (less or equal to 0.25) that are likely to be non-hemolytic examples. As we follow vertically, the second quadrant counted 272 AMPs with predicted low-moderate hemolytic probabilities and percentile outlier scores (light/baby blue circles, between 0.25 and 0.50). The third quadrant displayed 466 AMPs with predicted moderate-high hemolytic probabilities and percentile outlier scores (Maya blue circles, between 0.50 and 0.75). The fourth quadrant clustered 2036 AMPs with predicted high hemolytic probabilities and percentile outlier scores (blue circles, higher than 0.75). The fifth and last quadrant included 273 AMPs outliers across the entire range of hemolytic probabilities and percentile outlier scores (dark blue circles, higher than 0.99).

In Fig. 5b, we presented the distributions of representative AMP families according to their consensus class probabilities and (percentile) outlier scores. And, we screened for experimental validations of hemolytic (in)activity for each of the selected APD peptides through the Antimicrobial Peptide Database (https://aps.unmc.edu/AP/main.php). In the bottom left corner of the figure, we observed several amphibian dermaseptins (e.g. APD00162, APD00163, APD00942, APD00943, APD00963, APD01351, APD01352—lime green circles) and a pair of piscine hepcidicins (APD01701, APD01702—forest green circles), none of these peptides were tested against human erythrocytes^57,58^. Besides, miscellaneous AMPs from that cluster, i.e. the synthetic 27-residue fragment P27 of Seminalplasmin/APD00234^59^, Odorranain-M1/APD01300^60^, Ranatuerin-2PRb/APD01719^61^ and Ocellatin-PT6 /APD02734^62^ were reported not to exhibit hemolytic activity. The second quadrant included sequences of two AMP families derived from frog skin secretions; dermaseptins (in lime green) and brevinins (in turquoise) in addition to the four insect bactericidins or cecropin D-like peptides (APD00011, APD00032, APD00033, APD00034—orange circles). Most peptides mentioned above lacked experimental validation against red blood cells, only Brevinin-2-related peptide^63^ was mentioned as a low hemolytic peptide (APD00599: consensus class probability of 0.55 and percentile outlier score of 0.23). The third quadrant contained several toad-derived maximins (i.e. APD00062, APD00064, APD00065, APD01736—dark blue circles). One peptide APD01736 was found in the HAMP dataset while others were evaluated as low-moderate hemolytic peptides at 50 µg/mL^64^. The fourth quadrant gathered many cyclic plant AMPs known as cyclotides (in brown circles), which were common to the hemolytic dataset (HAMP). Also, this quadrant depicted two salmonid cathelicidins (APD02536, APD02539) and seven histone/histone-like proteins from different animal sources (APD00335, APD00337, APD00338, APD02804, APD02807, APD02808, APD02810) that were predicted with a wide range of consensus class probabilities. The salmonid cathelicidins, called rt CATH-1b and rtCATH-2a, were predicted with class probabilities of 0.34–0.35, and they did not exhibit hemolytic activity against trout and human erythrocytes at 60 µM^65^. None of the histone/histone-like proteins were tested for hemolytic activity. In the last quadrant, one outlying AMP family contained six neuropeptide-like proteins (NLPs) that originated from the nematode *Caenorhabditis Elegans* (i.e. APD01487-1492—pink circles)^66^. Most displayed antimicrobial properties against various pathogens; only one peptide NLP-31/APD01491 was evaluated as non-cytotoxic against mammalian cells^67^. Our models predicted this NLP with a high consensus class probability of 0.82. All APD indices, consensus class probabilities and outlier scores were provided in the Supplementary Table S8.

### Defining the characteristics of current hemolytic models outliers

**Figure 6:**
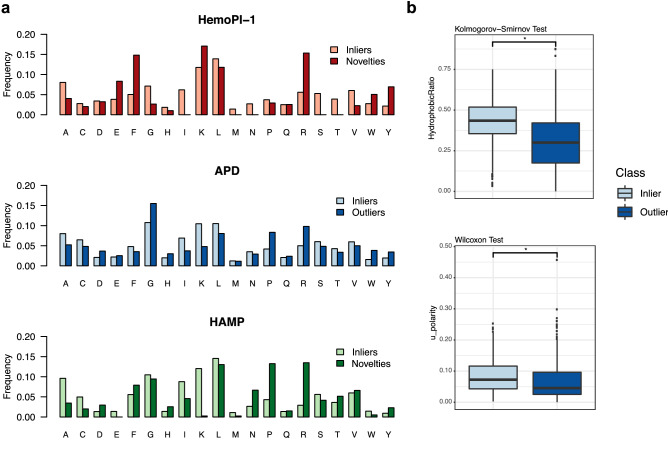
(**a**) Amino-acid compositions of inliers, novelties and outliers from 884 HemoPI-1 peptides (reds), 3081 APD peptides (blues), 317 HAMP peptides (greens). (**b**) Distributions of two physicochemical properties, Hydrophobic ratio (top) and moment of polarity (bottom) between two groups; APD inliers (light blue) and APD outliers (dark blue).

All multivariate outlier detection method, presented in Fig. 3, employed the 56 physicochemical properties to determine the outlier score. Therefore, one can think that some of these features distinguished inliers, novelties and outliers. To date, it is not possible to extract important features directly from multivariate outliers detection methods. Instead, we analysed the amino acid compositions (AAC) of inliers, novelties and outliers in all three datasets; HemoPI-1 (reds), APD (blues) and HAMP (greens), as illustrated in Fig. 6a. We observed that arginine, lysine, leucine and isoleucine were well represented across the 3 datasets. Arginine was particularly present among novelties and outliers. Glutamic acid and phenylalanine composed numerous HemoPI-1 novelties. APD outliers and HAMP novelties were rich in glycine and proline while their inliers consisted of alanine, lysine, leucine and isoleucine. Besides, we explored differences between physicochemical property distributions between APD inliers (2808) and APD outliers (273). For both groups, we measured all physicochemical properties and we evaluated the 112 distributions for normality, variance, false discovery rate and parametric or non-parametric tests. Most properties did not follow a normal distribution and had unequal variances, as summarized in Fig. S3. In Table S9, we outlined p-values and adjusted p-values (p-adjust) for all properties after applying the appropriate statistical tests (Welch, Wilcoxon or Kolmogorov–Smirnov). Finally, we depicted the physicochemical property distributions for both APD inliers and outliers in a series of 56 boxplots where we indicated significant differences with a p-value lower than 0.001 (*)—Fig. S4a–d. We found that many properties, indices or moments of hydrophobicity on different scales (Eisenberg, GRAVY, Janin, etc.), aromaticity and bulkiness, were statistically significant between the two groups. Additional features of polarity and flexibility also significantly differed between APD inliers and outliers. Figure 6b illustrated these differences by showing two significant properties; hydrophobic ratio (top) and moment of polarity (bottom). Comparing between medians, APD inliers were more hydrophobic by 10–15 points and more polar by 2–4 points than their outlying counterparts of the same length (Tables S10, S11). We attributed the greater hydrophobicity of APD inliers to their enrichment in the particular residues, phenylalanine (F), leucine (L) and isoleucine (I). Prevalent lysine (K), serine (S), cysteine (C), or arginine (N) led to more polar peptides (Fig. 6a). In contrast, APD outliers contained additional glycine (G) or proline (P), these residues are known to break secondary structures like helices and they could explain their greater flexibility (Fig. S4b). In Fig. S5, we reported the amino acid composition and the frequencies of certain residues (positively charged residues lysine, arginine, histidine; negatively charged aspartic and glutamic acids; small residues glycine, cysteine, alanine, proline, serine and bulky residues leucine, isoleucine, phenylalanine, tyrosine, tryptophan) within the 3081 sequences. Overall, most APDs with outlier scores higher than 0.8 had high frequencies in positively charged arginine (dark purple—Figure S5a), in small amino acids i.e., glycine, proline as well as some bulky residues i.e., tryptophan, tyrosine (dark purple and dark orange—Figure S5b).

### Designing de novo non-hemolytic AMP-like peptides

**Figure 7:**
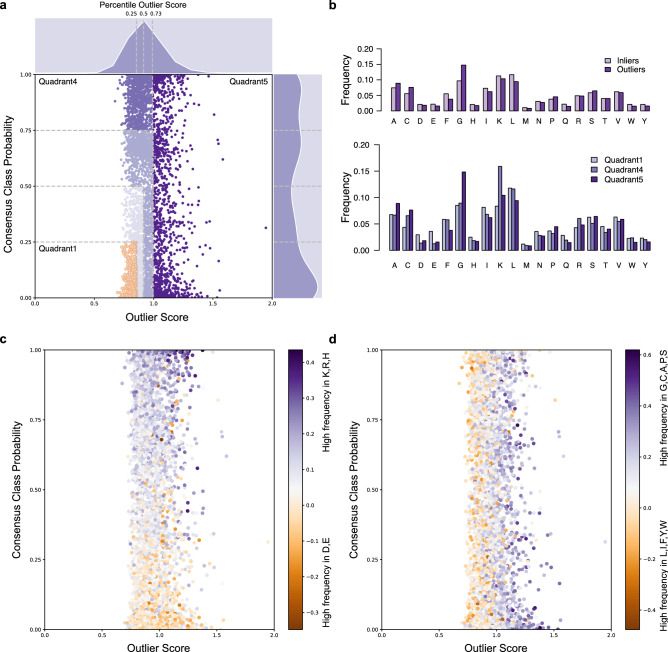
Scatterplots showing the distribution of 5000 generated inliers sequences GIS according to outlier scores and hemolytic (HemoPI-1) consensus class probabilities. (**a**) Changes in the outlier scores and probabilities are illustrated with different shades of purples. Selected 507 non-hemolytic generated sequences with lowest outlier scores and hemolytic predictions (golden circles, quadrant 1). Generated sequences with highest haemolytic predictions are in quadrant 4. Generated sequences identified as outliers according to HemoPI-1 Average KNN (darkest purple data points, quadrant 5). (**b**) Differences in amino acid composition between the 3 quadrants 1/4/5. (**c**) Changes in colour gradient indicate differential enrichment in positively charged amino acids i.e. lysine, arginine, histidine (purple) or negatively charged amino acids i.e. aspartic and glutamic acids (orange). (**d**) Changes in colour gradient indicate differential enrichment in small amino acids i.e. glycine, cysteine, alanine, proline, serine (purple) or bulky aromatic/aliphatic amino acids i.e. phenylalanine, tyrosine, tryptophan, leucine, isoleucine (orange).

The discovery of APD inliers with different hemolytic profiles provided the basis to design de novo new AMP-like peptides and to extrapolate over the characteristics (physicochemical properties, amino-acid composition) of novel non-hemolytic peptides. We generated de novo a library of 5000 random peptide sequences (RPS) sharing the lengths and the AA frequencies of 2808 APD inliers. We chose to generate this library from all the APD inliers, and not solely from the 34 non-hemolytic examples (golden circles, Fig. 5a), for the sake of diversity in terms of physicochemical properties, amino-acid composition, and plausible secondary structures. Our models predicted the hemolytic nature and outlier scores of newly designed peptides, the results were displayed in Fig. 7. Looking at Figs. 5a and 7a, we noted that the 5000 class probabilities were more balanced over the whole range, even slightly pulled down, than those of APD inliers. This difference in balance suggested an enrichment in AMP-like sequences of non-hemolytic nature, we counted 507 peptides (10.1%) versus 34 peptides (1.1%) in that quadrant. These generated sequences distributed normally across a comparable window of outlier scores, however, their mean value of outlier scores was closer to 1 (RPS: 0.92 vs. APD: 0.69) leading to a higher fraction of outliers. Average KNN identified 1271 (25.4%) outlying generated sequences compared to 273 APD outliers (8.9%). We examined more closely three of the five quadrants: the first quadrant (1) consisted of 507 RPS inliers with the lowest class probabilities and outlier scores, the second quadrant (4) contained 794 RPS inliers with high hemolytic predictions and the last quadrant (5) comprised the 1271 RPS outliers. We compared the amino-acid compositions of RPS inliers and outliers as well as between the selected quadrants, as illustrated in Fig. 7b. Like APD inliers, the 3729 RPS inliers were enriched in phenylalanine, lysine, leucine, isoleucine whereas RPS outliers consisted in small amino acids i.e., glycine, proline as well as serine, alanine, cysteine. Generated sequences of hemolytic nature (quadrant 4) included higher proportion of lysine than their non-hemolytic counterparts (quadrant 1). We believed that generating de novo random peptide sequences based on the amino acid composition of APD inliers served as a beacon to amplify the punctual observations made in Figure S5. We could not tell if the large number of RPS outliers resulted from the random generation.

In order to provide guidelines for the design of non-hemolytic peptides, we analysed the 5000 random peptide sequences for their contents in small, aromatic, aliphatic and charged amino acids across hemolytic predictions and outlier scores. We measured differences in cumulative frequencies between positively charged and negatively charged amino acids (Fig. 7c), and cumulative frequencies between small and bulky amino acids (Fig. 7d). Positively charged amino acids include lysine, arginine, histidine, and negatively charged amino acids consisted of aspartic and glutamic acids. Small amino acids were glycine, cysteine, alanine, proline, serine while bulky amino acids encompassed aromatic and aliphatic residues, i.e. phenylalanine, tyrosine, tryptophan, leucine, isoleucine. In Fig. 7c, cumulative differential frequencies indicated that higher hemolytic predictions correlated with a higher percentage in positively charged residues, particularly lysine and arginine. We estimated that these residues counted for 23.8 ± 5.1% of RPS in quadrant 4 versus 15.1 ± 4.3% of RPS in quadrant 1 (Fig. 7a, Table S12). Non-hemolytic peptide sequences could be either neutral or slightly charged with cumulative differential frequencies ranging between – 0.2 and + 0.1. The prevalence of negatively charged residues in RPS from quadrant 1 (6.5 ± 3.2%) compared to RPS from quadrants 4 (2.5 ± 2.6%) and 5 (3.4 ± 3.8%) could explain that net charge balance. RPS inliers contained as many bulky residues as small amino acids (28.6–30.4% RPS from quadrants 1 and 4—Table S12) where aromatic amino acids (i.e., F/W/Y) and aliphatic residues (i.e., L/I) represented 10 and 20% of the sequences. Finally, Table S12 showed that RPS outliers (quadrant 5) presented a more significant proportion of small amino acids (19.3 ± 7.5% G/P and 42.3 ± 9.1% G/C/A/P/S) compared with RPS inliers. In sum, these residues counted for 82–85% of the sequences, the rest was filled with amino acids of different types. Similar trend held for the 3081 sequences of APD dataset (Table S13).

## Discussion

The first part of our study described the development of predictive models for the discovery and design of non-hemolytic peptides. In the last five years, the number of online platforms for hemolytic activity prediction has risen; we identified HemoPred^18^, HemoPI^19^, HLPpred-Fuse^21^, HAPPENN^22^ and HemoPImod^68^. The latter predicted the hemolytic potency of chemically modified peptides and the last three services were published in 2020. For the sake of reproducibility and comparison with HemoPred^18^, HemoPI^19^ and HLPpred-Fuse^21^, we built our predictive models, binary classifiers, using the publicly available HemoPI-1, HemoPI-2 and HemoPI-3 datasets. Among the previously benchmarked algorithms, HemoPI designers identified support vector machine (SVM) as the best classifying algorithm while creators of HemoPred platform favoured random forest (RF/CART) classifier for its built-in estimation of feature importance. Both studies utilized the popular decision tree J48, RF and SVM using WEKA and R programs (RWeka package). Chaudhary et al*.* further added nearest neighbour IBK, logistic regression (LR), multilayer perceptron. For HLP_Fuse, Hasan and co-workers evaluated three additional tree-based algorithms, namely gradient boosting (GBC), adaptive boosting (ABC) and extreme randomized tree (ERT). Their best models, using ERT algorithm, outperformed the existing predictors HemoPred and HemoPI by 2.5–4.1 points in accuracy for its first-layer (identifying hemolytic peptides) and by 4.9–5.3 points (predicting hemolytic activity). In our study, gradient boosting and extreme gradient boosting classifiers outperformed other algorithms. Random forest and support vector algorithms did not perform as well as the aforementioned online predictors when used with 56 modlAMP physicochemical descriptors. We have further improved the performances and robustness of our models using dimensionality reduction techniques, i.e. multicollinearity and recursive feature elimination, and optimising specific hyperparameters. Our finest models, shown in Tables 2 and 3, predicted the hemolytic nature from any peptide sequence with 95–97% accuracy and its hemolytic activity at high or low concentration with 77–80% accuracy.

Three of the five existing predictors, i.e. HemoPred^18^, HemoPI^19^, HLPpred-Fuse^21^, used the three HemoPI datasets allowing direct comparison between model performances. We commonly reported accuracy (Acc.) and Matthews correlation coefficient (MCC). Our final models 1.2 and 1.3 identified hemolytic peptides (HemoPI-1 dataset) better than HemoPred (Acc. 94.3%, MCC 0.89) and HemoPI (Acc. 96.0%, MCC 0.91) but less than the first layer of HLPpred-Fuse (Acc. 98.4%, MCC 0.97). Likewise, our models 2.1–2.3 classified hemolytic peptides at high or low concentration (HemoPI-2 dataset) with 76.7–77.8% in accuracy and MCC values of 0.53–0.55 in the same order of magnitude as HemoPred (Acc. 76.2%, MCC 0.52) and HemoPI (Acc. 78.3%, MCC 0.56) but less than the second layer of HLPpred-Fuse (Acc. 79.2%, MCC 0.59). Our models 3.1–3.3 performed similarly to HemoPred (Acc. 77.2%, MCC 0.54) and HemoPI (Acc. 79.9%, MCC 0.59) with accuracy values of 78.0–80.0% and MCC values of 0.56–0.60. Overall, our models performed as the existing predictors HemoPred^55^, HemoPI^19^, HLPpred-Fuse^21^. Except for model 1.1, all of our leading binary classifiers grounded on the gradient boosting algorithm. Of note, Hasan and co-workers observed that tree-based algorithms RF, ERT or GBC similarly outperformed other classifiers in the presence of the specific amino acid encoding QSO^21^. Gradient boosting classification has a build-in estimation of feature importance; therefore, we determined which physicochemical properties contributed the most to the binary classifiers. We identified that size, shape, charge, polarity and hydrophobicity are the main properties that governed the hemolytic nature and activity of peptides. This observation was in agreement with previous thermodynamic studies^69, 70^ about peptides (and other macrocycles) known to recognise, interact with phospholipids and permeate through cell membranes. Applying our best models to 3081 AMPs from the Antimicrobial Peptide Database revealed that nearly 300 known hemolytic antimicrobial peptides (HAMP) were correctly predicted. Our models accurately assigned several non-hemolytic AMPs such as the synthetic 27-residue fragment P27 of Seminalplasmin/APD00234^59^, Odorranain-M1/APD01300^60^, Ranatuerin-2PRb/APD01719^61^, Ocellatin-PT6 /APD02734^62^, toad-derived Maximin 45/APD01736^64^ and the two salmonid cathelicidins rtCATH-1b/APD02535 and rtCATH-2a/APD02539^65^.

In order to strengthen the scientific validity our predictive models, we defined their applicability domain (AD) as recommended by the guidelines of the Organization for Economic Cooperation and Development (OECD)—Principle 3^71^. Each model dataset could be mapped onto a multidimensional space using N variables, e.g. 56 (or less) physicochemical properties. The domain of applicability represents the region of property space where the hemolytic predictions would be considered reliable. In the absence of an AD restriction, each model can predict the hemolytic nature and activity of any peptide sequence, which could be strictly different from those of model datasets, resulting in extrapolated and inaccurate predictions^72^. This task became all the more essential since none of the existing online servers for hemolytic activity prediction has established the limitations of their QSA/PR models. After a thorough review of the literature, we believed this is the first time that a study defined the applicability domain in peptide modelling leading to the identification of outlying sequences. We found that Zheng and co-workers recently reported the applicability domains of their in silico models for hemolytic toxicity prediction applied to small molecules and fragments by average similarity^73,74^. To establish the applicability domain of our binary classifiers, we delineated boundaries using unsupervised detection of univariate and multivariate outliers. Noteworthy, such an approach would benefit greatly to any model based on at least one of the three HemoPI datasets. For univariate outliers, we reduced our 56-dimensional datasets to a single dimension, i.e. Mahalanobis distance (MD)^41^ before identifying outliers by empirical rule. This method yielded 47 HemoPI-1, 23 HemoPI-2 and 50 HemoPI-3 novelties, representing 3–5% of total datasets. In our testing datasets, the number of novelties and outliers varied according to the model used for hemolytic predictions. Thus, we found from 4 to 52 novelties (1–16%) among 317 hemolytic AMPs (HAMP) and 89–440 outliers (3–14%) within 3081 peptides from APD (see Table S6). In addition to the detection of univariate novelties/outliers, we examined their detection in high-dimensional space, i.e. 56 physicochemical properties. Reducing any multivariate dataset into the single Mahalanobis distance to detect outliers might present disadvantages, e.g. the loss of information, that referred to as the “curse of dimensionality”^56^. We benchmarked 8 multivariate outlier detection (OD) methods; local outlier factor^42^, clustering-based local outlier factor^43^, histogram-based outlier score^44^, (Average) K-nearest neighbours^45^), isolation forest^46^, feature bagging^47^ and angle-based outlier detection^48^. Unlike MD, these multivariate OD methods are less rigid, resulting in an applicability domain with uneven limitations. For direct comparison with MD-guided detection, we used identical outlier fractions corresponding to 3–5% of total datasets. We assumed that the best outlier detector should encompass the maximum number of observations (inliers) from each HemoPI model dataset or it should pick the applicability domain with the lowest numbers of novelties (novel space) in a model property space. Average K-Nearest Neighbour (Average KNN) emerged as the best multivariate OD approach scoring the lowest number of novelties simultaneously across the property spaces of the three HemoPI datasets. That method yielded 14 HemoPI-1, 5 HemoPI-2 and 10 HemoPI-3 novelties, representing 0.6–1.6% of total datasets. HAMP novelties decreased to 3 (0.2–0.4%), and APD outliers varied between 253 and 273 (8.2–8.9%) in the applicability domains of HemoPI-based models (Table S7). Among APD outliers, we identified one AMP family that contained six neuropeptide-like proteins that originated from the nematode *Caenorhabditis Elegans*, i.e. APD01487-1492^66^; only one peptide NLP-31/APD01491 was evaluated as non-cytotoxic against mammalian cells^67^.

Beyond the discovery of novel (non-)hemolytic AMPs, we studied differences between peptide inliers and outliers intending to form guidelines for the design of non-hemolytic peptides. We evaluated their amino acid compositions in HemoPI-1, APD, and HAMP datasets. Arginine, lysine, leucine and isoleucine were well represented across the three datasets (Fig. 6). Arginine, glycine and proline were present among novelties and outliers. Glutamic acid and phenylalanine composed various HemoPI-1 novelties. Inliers with antimicrobial activity (APD, HAMP) consisted of alanine, lysine, leucine and isoleucine. Both Raghava and Nantasenamat groups^18,19^ previously reported as leucine, lysine, glycine, phenylalanine and arginine as the most frequent residues among hemolytic and non-hemolytic peptides. Win and co-workers also detailed the important roles that lysine, leucine and glycine may take to modulate the hemolytic nature and activity of a peptide as well as its amphipathic character^72^. We identified that APD inliers were overall more hydrophobic by 10–15 points and more polar by 2–4 points than their outlying counterparts of the same length. In 2020, Timmons and co-workers developed HAPPENN from 3738 experimentally validated peptides, the amino acid composition of that dataset concur with previous observations^22^. We suggested that hemolytic activity predictions made from peptide sequences particularly enriched in small amino acids (e.g. glycine, proline), charged residues (e.g. arginine, glutamic acid), and the aromatic tryptophan and tyrosine, may not be reliable. We further studied the importance of these residues by designing de novo a library of 5000 random peptide sequences (RPS) based on the amino acid composition of APD inliers. Nearly 500 novel sequences were predicted as non-hemolytic peptides. Our analyses in differential cumulative frequencies (Fig. 7) further supported the weights that small, bulky and charged amino acids played in predicting hemolytic activity and outliers. We concluded that to design non-hemolytic peptides; researchers should design neutral or slightly charged sequences with ~ 20% positively and negatively charged residues (ratio 3:1), an equal proportion (~ 30%) of aromatic/aliphatic residues (ratio 1:2) and small amino acids in random peptide sequences to insure robust hemolytic predictions.

## Conclusion

Peptides are taking an increasing share of the drug market. Peptide-based drug design faces many hurdles on the way to clinical trials including metabolic instability, poor oral bioavailability and toxicity. Machine learning models are cost-effective and time-saving strategies that have the potential to alleviate these hurdles and accelerate the selection of most promising peptide sequences from sizable libraries. The present study focused on predicting hemolytic nature and activity of peptides. Our gradient boosting classifiers outperformed existing online services for hemolytic activity prediction. Following OCDE guidelines, we defined the applicability domains of our predictive models using multivariate outlier detection methods, a first in QSA/PR modelling. Average KNN appeared like the method of choice to maximizing inliers (applicability domain) or minimize the number of outliers for hemolytic datasets (HemoPIs and HAMP). Such a method should be implemented into the existing predictors using HemoPI datasets, to avoid extrapolated predictions upon newly designed sequences. Our robust models were applied to 3081 antimicrobial peptides (AMPs), natural and synthetic peptides offering promising avenues against antibiotic-resistant infections. Most AMPs present in clinical trials are administrated topically due to their hemolytic toxicity. Predicting their hemolytic activity early in the drug discovery pipeline saves costs in biological testing, leaps to medicinal chemistry optimization. Nearly 30% of AMPs were predicted as non-hemolytic peptides, and about 91% of the predictions would be considered reliable. To design non-hemolytic random peptides, one should consider neutral or slightly charged sequences with an equal proportion of aromatic/aliphatic residues and small amino acids (or slightly more bulky amino acids).

## Supplementary information

Supplementary file1.

Supplementary file2.

## Data Availability

Supporting data in this article are provided in Supporting Information. Python and R scripts can be downloaded at https://github.com/plissonf/ML-guided-discovery-and-design-of-non-hemolytic-peptides.
