# Supplementary material for: Machine learning-guided discovery and design of non-hemolytic peptides
Source: Sci Rep. 2020 Oct 6;10:16581. doi: 10.1038/s41598-020-73644-6 (PMC7538962; doi:10.1038/s41598-020-73644-6)
Supplement: Supplementary file 1 — Supplementary file1. [file 41598_2020_73644_MOESM1_ESM.pdf]

Machine learning-guided discovery and design of non-hemolytic peptides

Fabien Plisson[1,*], Obed Ramírez-Sánchez[1], Cristina Martínez-Hernández[1]

[1]*CONACYT, Centro de Investigación y de Estudios Avanzados del IPN, Unidad de Genómica Avanzada, Laboratorio Nacional de Genómica para la Biodiversidad (Langebio), Irapuato, Guanajuato 36824, México.*

[*] Fabien Plisson; fabien.plisson@cinvestav.mx

Table of Contents

Abbreviations 3

List S1. Physicochemical descriptors (56) 4

Table S1a. Performance metrics of 13 binary classifiers for predicting hemolytic activity using HemoPI-1 model/validation datasets and 56 sequence-based physicochemical descriptors. 5

Table S1b. Performance metrics of 13 binary classifiers for predicting hemolytic activity using HemoPI-2 model/validation datasets and 56 sequence-based physicochemical descriptors. 6

Table S1c. Performance metrics of 13 binary classifiers for predicting hemolytic activity using HemoPI-3 model/validation datasets and 56 sequence-based physicochemical descriptors. 7

Table S2. Mean accuracies of top binary classifiers for the 3 different model/validation datasets (HemoPI-1, HemoPI-2, HemoPI-3) using optimal number of physicochemical descriptors after applying feature reduction methods: multicollinearity, recursive feature extraction and backward elimination. 8

Table S3. Mean accuracies (%) of top performing Gradient Boosting Classifier (GBC) for model/validation datasets (HemoPI-1, HemoPI-2, HemoPI-3) after reducing the number of features (N: physicochemical descriptors) using multicollinearity and correlation thresholds (0.75-0.95). 9

Table S4a. Performance metrics of leading binary classifiers for HemoPI-1 model/validation datasets after optimizing their respective hyperparameters with full or reduced number of features (multicollinearity, recursive feature extraction). 10

Table S4b. Performance metrics of leading binary classifiers for HemoPI-2 model/validation datasets after optimizing their respective hyperparameters with full or reduced number of features (multicollinearity, recursive feature extraction). 11

Table S4c. Performance metrics of leading binary classifiers for HemoPI-3 model/validation datasets after optimizing their respective hyperparameters with full or reduced number of features (multicollinearity, recursive feature extraction). 12

Table S5a. Performance metrics of binary classifier Extreme Gradient Boosting (XGBC) for HemoPI datasets with full or reduced number of features (multicollinearity, recursive feature elimination) using default model hyperparameters. 13

Table S5b. Performance metrics of binary classifier Extreme Gradient Boosting (XGBC) for HemoPI datasets with full or reduced number of features (multicollinearity, recursive feature elimination) using optimised model hyperparameters. 14

Table S6. Inliers, novelties (model datasets) and outliers (APD and HAMP datasets) counts applying empirical rule to Mahalanobis distances for HemoPI datasets. 15

Table S7. Inliers, novelties and outliers counts applying multivariate outlier detection methods to 56-dimensional datasets HemoPI-1, HemoPI-2, HemoPI-3, Antimicrobial Peptide Dataset (APD, 3081) and known haemolytic antimicrobial peptides in APD (HAMP, 317). 16

Table S8. Consensus class probabilities and outlier scores of APD dataset (additional spreadsheet)

Table S9. Statistical values (p-value and p-adjust) for all 56 physicochemical properties. 17

Table S10. Descriptive statistics for 56 physicochemical property distributions (APD inliers). 19

Table S11. Descriptive statistics for 56 physicochemical property distributions (APD outliers). 21

Table S12. Amino acid composition for specific random peptide sequences (RPS). 23

Table S13. Amino acid compositions for APD and RPS datasets. 23

Figure S1a. Feature Importances of top 3 binary classifiers for HemoPI-1 dataset. 24

Figure S1b. Feature Importances of top 3 binary classifiers for HemoPI-2 dataset. 25

Figure S1c. Feature Importances of top 3 binary classifiers for HemoPI-3 dataset. 26

Figure S2. Quality curves RNX(K) report the relative improvement over 17 different embedding techniques for HemoPI-1 model dataset. 27

Figure S3. Statistical studies to identify differences between the distributions of 56 physicochemical properties from two groups; APD inliers and outliers. 28

Figure S4a. Boxplots comparing the distributions of physicochemical properties 1-16 within inliers (cyan) and outliers (dark blue) groups. 29

Figure S4b. Boxplots comparing the distributions of physicochemical properties 17-32 within inliers (cyan) and outliers (dark blue) groups. 30

Figure S4c. Boxplots comparing the distributions of physicochemical properties 33-48 within inliers (cyan) and outliers (dark blue) groups. 31

Figure S4d. Boxplots comparing the distributions of physicochemical properties 49-56 within inliers (cyan) and outliers (dark blue) groups. 32

Figure S5. Scatterplots showing the distribution of 3,081 antimicrobial peptides (APD dataset) according to outlier scores and hemolytic (HemoPI-1) consensus class probabilities. 33

Abbreviations

LOGREG	Logistic Regression
KNN	K-Nearest Neighbour
CART	Classification and Regression (Decision) Tree
RFC	Random Forest Classifier
GBC	Gradient Boosting Classifier
ADC	Adaboost Classifier
LDA	Linear Discriminant Analysis
QDA	Quadratic Discriminant Analysis
NB	Naïve Bayes
SVC-LIN	Support Vector Classifier, linear kernel
SVC-RBF	Support Vector Classifier, radial basis function kernel
SVC-POLY	Support Vector Classifier, polynomial kernel
SVC-SIG	Support Vector Classifier, sigmoid kernel
XGBC	Extreme Gradient Boosting Classifier
Acc.	Accuracy
Prec.	Precision
MCC	Matthews Correlation Coefficient
CK	Cohen's kappa
AUC ROC	Area under curve Receiver Operating Characteristic
ABOD	Angle-based Outlier Detector
CBLOF	Cluster-based Local Outlier Factor
FB	Feature Bagging
HBOS	Histogram-Based Outlier Detector
IF	Isolation Forest
KNN	K-Nearest Neighbour
LOF	Local Outlier Factor
t-SNE	t-distributed Stochastic Neighbour Embedding

For all following results, each dataset was divided into a model set (80%) and an external validation set (20%), and both were subjected to stratified 10-fold cross-validation.

List S1. Physicochemical descriptors (56) – first 9 global descriptors and 47 peptide descriptors. Implementation using Python modules and references for all descriptors at https://modlamp.org/modlamp.html#module-modlamp.descriptors

Aromaticity: calculated global aromaticity, relative frequency of Phe+Trp+Tyr
AliphaticIndex: calculated aliphatic index of a sequence, a measure of thermal stability using amino acids Ala, Ile, Leu, Val
BomanIndex: calculated Boman index, a measure for protein-protein interactions
HydrophobicRatio: calculated hydrophobic ratio, relative frequency of Ala, Cys, Phe, Ile, Leu, Met and Val.
InstabilityIndex: calculated peptide stability from a sequence,
Length: peptide length
MW: calculated molecular weight of a sequence
NetCharge: calculated charge at pH 7.4 for a sequence
IsoelectricPoint: calculated isoelectric point of a sequence
B_Bulkiness: index with amino acid side chain bulkiness scale
uB_Bulkiness: moment with amino acid side chain bulkiness scale
charge_phys: amino acid charge at pH 7.0 - Hystidine charge +0.1.
charge_acid: amino acid charge at acidic pH - Hystidine charge +1.0
Ez: potential that assesses energies of insertion of amino acid side chains into lipid bilayers
F_Levitt: index with Levitt amino acid alpha-helix propensity scale
uF_Levitt: moment with Levitt amino acid alpha-helix propensity scale
flexibility: index with amino acid side chain flexibility scale
u_flexibility : moment with amino acid side chain flexibility scale
Grantham: index for amino acid side chain composition, polarity and molecular volume
H_argos: hydrophobicity index with Argos hydrophobicity amino acid scale
uH_argos: hydrophobic moment with Argos hydrophobicity amino acid scale
H_Eisenberg: hydrophobicity index with Eisenberg hydrophobicity amino acid scale
uH_Eisenberg: hydrophobic moment with Eisenberg hydrophobicity amino acid scale
H_GRAVY: hydrophobicity index with GRAVY hydrophobicity amino acid scale
uH_GRAVY: hydrophobic moment with GRAVY hydrophobicity amino acid scale
H_HoppWoods: hydrophobicity index with Hopp-Woods hydrophobicity amino acid scale
uH_HoppWoods: hydrophobic moment with Hopp-Woods hydrophobicity amino acid scale
H_Janin: hydrophobicity index with Janin hydrophobicity amino acid scale
uH_Janin: hydrophobic moment with Janin hydrophobicity amino acid scale
H_KyteDoolittle: hydrophobicity index with Kyte & Doolittle hydrophobicity amino acid scale
uH_KyteDoolittle: hydrophobic moment with Kyte & Doolittle hydrophobicity amino acid scale
ISAECI: index for Isotropic Surface Area (ISA) and Electronic Charge Index (ECI) of amino acid side chains
modlabs_ABHPRK: modlabs inhouse physicochemical feature scale (Acidic, Basic, Hydrophobic, Polar, aRomatic, Kink-inducer)
MSS_shape: a graph-theoretical index that reflects topological shape and size of amino acid side chains
u_MSS_shape moment of a graph-theoretical index that reflects topological shape and size of amino acid side chains
MSW: amino acid scale based on a PCA of the molecular surface based WHIM descriptor (MS-WHIM), extended to natural amino acids
pepArc: modlabs pharmacophoric feature scale, dimensions are: hydrophobicity, polarity, positive charge, negative charge, proline.
pepcats: modlabs pharmacophoric feature based PEPCATS scale
polarity: index using amino acid polarity scale
u_polarity: moment with amino acid polarity scale
PPCALI: modlabs inhouse scale derived from a PCA of 143 amino acid property scales
refractivity: relative amino acid refractivity values
u_refractivity: relative amino acid refractivity moment
S_AASI: index with an amino acid selectivity index scale for helical antimicrobial peptides
uS_AASI: moment with an amino acid selectivity index scale for helical antimicrobial peptides
t_scale: global t scale, A PCA derived scale based on amino acid side chain properties calculated with 6 different probes of the GRID program
TM_tend: index with amino acid transmembrane propensity scale
u_TM_tend: moment with amino acid transmembrane propensity scale moment
Z3_1, **Z3_2**, **Z3_3**: original three dimensional Z-scale
Z5_1, **Z5_2**, **Z5_3**, **Z5_4**, **Z5_5**: extended five dimensional Z-scale

HemoPI-1	Model (442/442)					Validation (110/110)				
Classifiers	Acc. (%)	Prec. (%)	MCC	CK	AUC ROC	Acc. (%)	Prec. (%)	MCC	CK	AUC ROC
LOGREG	92.6	89.4	0.853	0.853	0.926	89.6	84.7	0.792	0.791	0.896
KNN	93.2	89.6	0.865	0.864	0.932	86.8	81.1	0.738	0.736	0.868
CART	90.4	85.8	0.808	0.808	0.904	83.7	77.6	0.674	0.673	0.837
RFC	93.8	91.1	0.876	0.876	0.938	87.3	81.8	0.747	0.746	0.873
GBC	94.0	91.3	0.880	0.880	0.940	90.4	86.5	0.809	0.809	0.905
ADC	93.8	90.7	0.876	0.876	0.938	91.4	87.4	0.828	0.828	0.914
LDA	94.2	91.1	0.885	0.885	0.943	90.5	87.1	0.809	0.809	0.905
QDA	91.5	88.3	0.830	0.830	0.915	85.5	83.8	0.727	0.709	0.854
NB	85.9	82.3	0.721	0.717	0.859	85.5	81.0	0.710	0.709	0.855
SVC-LIN	88.1	84.2	0.763	0.762	0.881	85.0	81.1	0.703	0.700	0.850
SVC-RBF	88.7	84.8	0.774	0.774	0.887	85.5	81.0	0.710	0.709	0.855
SVC-POLY	71.5	71.4	0.521	0.430	0.715	60.0	57.0	0.232	0.200	0.600
SVC-SIG	88.0	84.1	0.761	0.760	0.880	81.8	79.2	0.655	0.636	0.818

Table S1a. Performance metrics of 13 binary classifiers for predicting hemolytic activity using HemoPI-1 dataset and 56 sequence-based physicochemical descriptors. **Acc.** mean accuracy, **Prec.** mean precision, **MCC** Matthews correlation coefficient, **CK** Cohen’s kappa, **AUC ROC** Area under curve Receiver Operating Characteristic.

HemoPI-2	Model (442/370)					Validation (110/92)				
**Classifiers**	**Acc. (%)**	**Prec. (%)**	**MCC**	**CK**	**AUC ROC**	**Acc. (%)**	**Prec. (%)**	**MCC**	**CK**	**AUC ROC**
LOGREG	68.0	65.2	0.350	0.348	0.672	65.8	63.2	0.305	0.298	0.646
KNN	71.6	68.1	0.423	0.421	0.708	63.4	61.5	0.253	0.248	0.621
CART	69.6	67.0	0.386	0.386	0.693	62.4	61.9	0.246	0.246	0.623
RFC	69.0	66.0	0.370	0.368	0.682	63.9	62.1	0.265	0.262	0.629
GBC	76.7	72.9	0.529	0.528	0.763	72.3	68.9	0.438	0.437	0.717
ADC	71.3	68.1	0.419	0.418	0.708	73.8	70.2	0.469	0.468	0.732
LDA	70.0	66.6	0.391	0.387	0.691	59.9	59.7	0.186	0.185	0.592
QDA	66.4	64.5	0.322	0.322	0.661	60.9	60.5	0.208	0.208	0.604
NB	62.3	61.7	0.243	0.243	0.622	63.4	62.0	0.257	0.256	0.627
SVC-LIN	61.9	59.7	0.230	0.199	0.595	59.9	57.8	0.210	0.137	0.564
SVC-RBF	63.7	61.2	0.262	0.244	0.618	62.4	59.7	0.252	0.200	0.595
SVC-POLY	54.4	54.4	0.000	0.000	0.500	54.5	54.5	0.000	0.000	0.500
SVC-SIG	59.6	57.7	0.195	0.133	0.563	54.5	54.5	0.000	0.000	0.500

Table S1b. Performance metrics of 13 binary classifiers for predicting hemolytic activity using HemoPI-2 dataset and 56 sequence-based physicochemical descriptors. **Acc.** mean accuracy, **Prec.** mean precision, **MCC** Matthews correlation coefficient, **CK** Cohen's kappa, **AUC ROC** Area under curve receiver operating characteristic.

HemoPI-3	Model (708/590)					Validation (177/148)				
**Classifiers**	**Acc. (%)**	**Prec. (%)**	**MCC**	**CK**	**AUC ROC**	**Acc. (%)**	**Prec. (%)**	**MCC**	**CK**	**AUC ROC**
LOGREG	69.7	66.1	0.386	0.377	0.685	70.8	67.0	0.408	0.40.0	0.697
KNN	72.8	69.3	0.449	0.447	0.722	65.8	63.4	0.305	0.30.1	0.648
CART	68.9	66.6	0.373	0.373	0.686	64.0	62.7	0.273	0.273	0.636
RFC	72.4	68.3	0.443	0.433	0.713	66.8	64.1	0.325	0.320	0.658
GBC	75.8	71.9	0.510	0.508	0.752	72.9	69.4	0.452	0.450	0.724
ADC	72.3	68.6	0.438	0.435	0.715	71.4	68.1	0.420	0.419	0.708
LDA	70.8	66.9	0.410	0.399	0.696	70.8	67.3	0.407	0.403	0.699
QDA	65.9	64.0	0.309	0.309	0.654	59.4	59.1	0.172	0.169	0.583
NB	64.4	62.8	0.278	0.277	0.638	65.9	64.0	0.309	0.309	0.654
SVC-LIN	65.4	62.0	0.309	0.272	0.630	66.5	62.8	0.330	0.298	0.643
SVC-RBF	66.3	62.7	0.325	0.292	0.641	66.1	62.8	0.319	0.294	0.640
SVC-POLY	54.6	54.6	0.000	0.000	0.500	54.5	54.6	0.000	0.000	0.500
SVC-SIG	63.4	60.4	0.272	0.224	0.606	63.4	60.3	0.280	0.222	0.605

Table S1c. Performance metrics of 13 binary classifiers for predicting hemolytic activity using HemoPI-3 dataset and 56 sequence-based physicochemical descriptors. **Acc.** mean accuracy, **Prec.** mean precision, **MCC** Matthews correlation coefficient, **CK** Cohen's kappa, **AUC ROC** Area under curve receiver operating characteristic.

Classifier	Mean Accuracy (%)								
	HemoPI-1 dataset			HemoPI-2 dataset			HemoPI-3 dataset		
	N	Model	Validation	N	Model	Validation	N	Model	Validation
Multicollinearity (threshold = 0.75)									
LOGREG	26	92.8	86.4	27	66.3	66.3	28	69.6	70.5
RFC	26	92.3	85.5	27	74.0	61.9	28	73.2	66.5
GBC	**26**	**95.4**	**91.8**	**27**	**77.5**	**71.3**	**28**	**76.2**	**72.0**
LDA	26	93.4	90.0	27	68.4	63.4	28	70.3	72.0
SVC-RBF	26	89.6	84.6	27	64.2	62.9	28	65.3	66.5
Recursive Feature Elimination (RFECV)									
RFC	34	94.9	90.0	34	72.8	69.8	22	75.0	69.2
GBC	31	95.0	90.5	**15**	**77.8**	**73.3**	**55**	**75.7**	**72.0**
LDA	**18**	**95.1**	**94.5**	53	70.0	59.9	40	71.1	68.9
Backward Elimination (BE)									
RFC	23	93.7	85.9	16	74.9	64.9	18	74.4	65.9
GBC	23	94.5	89.1	16	74.3	68.8	18	74.4	69.2
LDA	23	94.1	93.6	16	70.1	65.4	18	72.0	70.2

Table S2. Mean accuracies (%) of top binary classifiers using the different model datasets (HemoPI-1, HemoPI-2, HemoPI-3). Classifiers are Logistic Regression (LOGREG), Random Forest (RFC), Gradient Boosting (GBC), Linear Discriminant Analysis (LDA) and Support Vector machine with radial basis function kernel (SVC-RBF) after applying 3 feature reduction methods; 10-fold cross-validation recursive feature elimination, backward elimination and multicollinearity and N, final number of features.

	**Model**					**Validation**				
Corr. threshold	0.95	0.90	0.85	0.80	**0.75**	0.95	0.90	0.85	0.80	**0.75**
HemoPI-1 dataset										
N descriptors	50	42	35	31	**26**	50	42	35	31	**26**
GBC	94.9	95.2	95.2	95.5	**95.1**	89.6	90.4	90.1	89.1	**91.8**
HemoPI-2 dataset										
N descriptors	51	44	36	32	**27**	51	44	36	32	**27**
GBC	74.6	75.4	75.3	75.4	**77.5**	67.8	69.3	72.3	71.3	**71.3**
HemoPI-3 dataset										
N descriptors	51	43	36	33	**28**	51	43	36	33	**28**
GBC	76.0	74.4	74.6	74.5	**76.2**	72.3	71.7	71.7	68.0	**72.0**

Table S3. Mean accuracies (%) of top performing Gradient Boosting Classifier for model/validation datasets (HemoPI-1, HemoPI-2, HemoPI-3) after reducing the number of features (N: physicochemical descriptors) using multicollinearity and correlation thresholds ranging from 0.75 to 0.95. Optimal correlation thresholds were selected using best overall model and validation accuracies (in bold).

Classifier	Parameters	N	Acc. (%)	Prec. (%)	MCC	CK	AUC ROC
All 56 descriptors							
LOGREG	'C': 1000, 'penalty': 'l2', 'solver': 'lbfgs', 'tol': 0.1	56	94.9	92.6	0.898	0.898	0.949
			86.4	81.7	0.727	0.727	0.864
RFC	'max_depth': 10, 'max_features': 'sqrt', 'min_samples_leaf': 4, 'n_estimators': 128	56	95.6	93.6	0.912	0.912	0.956
			88.6	84.6	0.773	0.773	0.886
GBC	'max_depth': 4, 'max_features': 'sqrt', 'min_samples_leaf': 10, 'n_estimators': 208	56	96.0	94.6	0.921	0.921	0.960
			92.3	89.2	0.846	0.846	0.923
LDA	'solver': 'svd', 'tol': 0.1	56	94.7	91.8	0.894	0.783	0.947
			89.1	84.8	0.782	0.782	0.891
SVC-RBF	'C': 10, 'gamma': 0.1	56	95.5	93.4	0.909	0.909	0.955
			90.9	87.2	0.818	0.818	0.909
Multicollinearity (threshold = 0.75)							
LOGREG	'C': 1000, 'penalty': 'l2', 'solver': 'newton-cg', 'tol': 0.1	26	94.0	91.2	0.880	0.880	0.940
			89.6	85.9	0.791	0.791	0.896
RFC	'max_depth': 14, 'max_features': 'log2', 'min_samples_leaf': 2, 'n_estimators': 208	26	94.8	92.6	0.896	0.896	0.948
			89.1	85.1	0.782	0.782	0.891
GBC	'max_depth': 4, 'max_features': 'sqrt', 'min_samples_leaf': 10, 'n_estimators': 240	26	96.5	95.0	0.930	0.930	0.965
			92.7	89.6	0.855	0.855	0.927
LDA	'solver': 'svd', 'tol': 0.0001	26	93.4	90.2	0.869	0.869	0.934
			90.0	85.7	0.800	0.800	0.900
SVC-RBF	'C': 1, 'gamma': 1	26	95.1	93.5	0.903	0.903	0.951
			87.3	82.0	0.746	0.746	0.873
Recursive Feature Elimination (RFECV)							
RFC	'max_depth': 6, 'max_features': 'log2', 'min_samples_leaf': 4, 'n_estimators': 48	51	95.2	93.0	0.905	0.905	0.952
			90.9	87.2	0.818	0.818	0.909
GBC	'max_depth': 16, 'max_features': 'sqrt', 'min_samples_leaf': 10, 'n_estimators': 32	25	95.1	92.6	0.903	0.903	0.951
			90.0	86.3	0.800	0.800	0.900
LDA	'solver': 'svd', 'tol': 0.0001	18	95.1	92.6	0.903	0.903	0.951
			94.6	92.5	0.891	0.891	0.946

Table S4a. Performance metrics of binary classifiers Logistic Regression (LOGREG), Random Forest (RFC), Gradient Boosting (GBC), Linear Discriminant Analysis (LDA) and Support Vector machine with radial basis function kernel (SVC-RBF) using HemoPI-1 model/validation datasets after optimizing their respective hyperparameters with full or reduced number of features (N: physicochemical descriptors).

Classifier	Parameters	N	Acc. (%)	Prec. (%)	MCC	CK	AUC ROC
All 56 descriptors							
LOGREG	'C': 1000, 'penalty': 'l2', 'solver': 'newton-cg', 'tol': 0.1	56	70.7	67.5	0.406	0.405	0.701
			60.9	60.4	0.207	0.207	0.603
RFC	'max_depth': 16, 'max_features': 'log2', 'min_samples_leaf': 2, 'n_estimators': 144	56	76.9	73.2	0.532	0.532	0.765
			69.8	66.1	0.389	0.380	0.686
GBC	'max_depth': 4, 'max_features': 'sqrt', 'min_samples_leaf': 2, 'n_estimators': 112	56	77.7	74.0	0.549	0.549	0.774
			74.3	70.4	0.479	0.476	0.736
LDA	'solver': 'svd', 'tol': 0.2	56	68.5	65.5	0.360	0.356	0.676
			63.4	61.9	0.256	0.255	0.626
SVC-RBF	'C': 1000, 'gamma': 0.1	56	77.7	74.2	0.550	0.550	0.774
			65.3	63.9	0.303	0.303	0.652
Multicollinearity (threshold = 0.75)							
LOGREG	'C': 100, 'penalty': 'l2', 'solver': sag, 'tol': 0.1	27	67.3	64.6	0.335	0.332	0.664
			61.9	61.0	0.226	0.225	0.612
RFC	'max_depth': 18, 'max_features': log2, 'min_samples_leaf': 2, 'n_estimators': 48	27	76.7	73.1	0.530	0.530	0.764
			71.3	67.9	0.418	0.416	0.706
GBC	'max_depth': 18, 'max_features': None, 'min_samples_leaf': 8, 'n_estimators': 80	27	78.6	74.9	0.567	0.567	0.783
			71.3	67.7	0.418	0.414	0.704
LDA	'solver': 'svd', 'tol': 0.0001	27	68.4	65.3	0.358	0.354	0.675
			63.4	62.0	0.257	0.256	0.627
SVC-RBF	'C': 1000, 'gamma': 1	27	72.1	69.3	0.437	0.437	0.719
			69.8	67.3	0.391	0.391	0.695
Recursive Feature Elimination (RFECV)							
RFC	'max_depth': 14, 'max_features': None, 'min_samples_leaf': 2, 'n_estimators': 128	27	77.8	74.1	0.552	0.552	0.775
			70.8	67.2	0.408	0.403	0.699
GBC	'max_depth': 4, 'max_features': None, 'min_samples_leaf': 10, 'n_estimators': 128	54	77.7	73.9	0.549	0.549	0.773
			69.8	66.7	0.388	0.385	0.691
LDA	'solver': 'svd', 'tol': 0.1	48	69.6	66.3	0.383	0.379	0.687
			60.9	60.2	0.205	0.204	0.601

Table S4b. Performance metrics of binary classifiers Logistic Regression (LOGREG), Random Forest (RFC), Gradient Boosting (GBC) and Support Vector machine with radial basis function kernel (SVC-RBF) using HemoPI-2 model/validation datasets after optimizing their respective hyperparameters with full or reduced number of features (N: physicochemical descriptors).

Classifier	Parameters	N	Acc. (%)	Prec. (%)	MCC	CK	AUC ROC
All 56 descriptors							
LOGREG	'C': 1000, 'penalty': 'l2', 'solver': 'newton-cg', 'tol': 0.0001	56	70.9	67.2	0.410	0.403	0.698
			70.8	67.5	0.408	0.406	0.701
RFC	'max_depth': 20, 'max_features': 'log2', 'min_samples_leaf': 2, 'n_estimators': 176	56	78.0	74.0	0.554	0.553	0.775
			70.2	66.8	0.395	0.391	0.693
GBC	'max_depth': 18, 'max_features': 'log2', 'min_samples_leaf': 10, 'n_estimators':192	56	80.0	76.4	0.597	0.597	0.796
			71.7	68.3	0.427	0.425	0.711
LDA	'solver': 'svd', 'tol': 0.0001	56	70.8	66.9	0.410	0.399	0.696
			70.8	67.2	0.407	0.403	0.699
SVC-RBF	'C': 10, 'gamma': 0.1	56	73.0	68.4	0.458	0.442	0.716
			72.0	68.0	0.434	0.426	0.710
Multicollinearity (threshold = 0.75)							
LOGREG	'C': 1, 'penalty': 'l2', 'solver': 'newton-cg', 'tol': 0.2	28	69.9	66.2	0.390	0.381	0.687
			70.8	67.1	0.408	0.402	0.698
RFC	'max_depth': 12, 'max_features': 'sqrt', 'min_samples_leaf': 2, 'n_estimators': 96	28	76.4	72.6	0.523	0.521	0.759
			71.7	68.0	0.426	0.422	0.709
GBC	'max_depth': 20, 'max_features': 'log2', 'min_samples_leaf': 8, 'n_estimators': 96	28	78.0	74.4	0.556	0.556	0.777
			72.6	69.0	0.445	0.443	0.719
LDA	'solver': 'svd', 'tol': 0.2	28	70.3	66.5	0.400	0.390	0.703
			75.4	70.9	0.503	0.497	0.745
SVC-RBF	'C': 10, 'gamma': 1	28	71.7	67.4	0.429	0.415	0.703
			72.0	67.9	0.434	0.426	0.709
Recursive Feature Elimination (RFECV)							
RFC	'max_depth': 18, 'max_features': 'sqrt', 'min_samples_leaf': 2, 'n_estimators': 160	32	77.6	73.5	0.546	0.544	0.770
			72.0	68.3	0.433	0.429	0.712
GBC	'max_depth': 12, 'max_features': 'sqrt', 'min_samples_leaf': 8, 'n_estimators': 160	40	78.2	74.4	0.559	0.558	0.777
			74.5	70.8	0.483	0.482	0.740
LDA	'solver': 'svd', 'tol': 0.0001	16	71.4	67.2	0.425	0.410	0.700
			72.0	67.9	0.434	0.426	0.709

Table S4c. Performance metrics of binary classifiers Logistic Regression (LOGREG), Random Forest (RFC), Gradient Boosting (GBC) and Support Vector machine with radial basis function kernel (SVC-RBF) using HemoPI-3 model/validation datasets after optimizing their respective hyperparameters with full or reduced number of features (N: physicochemical descriptors) using multicollinearity threshold of 0.75 and 10-fold cross-validation recursive feature elimination.

Parameters	Feature Reduction	N	Acc. (%)	Prec. (%)	MCC	CK	AUC ROC
HemoPI-1 model and validation datasets							
default	none	56	94.8	92.4	0.896	0.896	0.948
			87.7	83.0	0.755	0.755	0.877
default	MC (0.75)	26	95.7	93.7	0.914	0.914	0.957
			92.3	88.5	0.846	0.846	0.923
default	RFECV	18	95.3	93.1	0.905	0.905	0.953
			89.1	84.8	0.782	0.782	0.891
HemoPI-2 model and validation datasets							
default	none	56	76.1	72.3	0.517	0.517	0.756
			69.3	66.4	0.378	0.378	0.687
default	MC (0.75)	27	76.1	72.4	0.517	0.516	0.757
			69.8	66.4	0.388	0.383	0.689
default	RFECV	51	74.5	70.9	0.484	0.484	0.741
			69.3	66.6	0.379	0.378	0.688
HemoPI-3 model and validation datasets							
default	none	56	74.7	71.1	0.487	0.486	0.741
			73.2	69.9	0.459	0.458	0.728
default	MC (0.75)	28	75.0	71.4	0.495	0.494	0.746
			72.0	68.6	0.433	0.431	0.714
default	RFECV	23	76.3	72.5	0.520	0.518	0.758
			72.3	69.1	0.440	0.439	0.719

Table S5a. Performance metrics of binary classifier Extreme Gradient Boosting for HemoPI datasets with full or reduced number of features (multicollinearity, recursive feature elimination) using default model hyperparameters.

Parameters	Feature Reduction	N	Acc. (%)	Prec. (%)	MCC	CK	AUC ROC
HemoPI-1 model and validation datasets							
'colsample_bytree': 0.9, 'eta': 0.1, 'max_depth': 10, 'min_child_weight': 1, 'subsample': 0.7, 'tree_method': 'hist', 'objective':'binary:logistic'	none	56	95.5	93.4	0.909	0.909	0.955
			88.6	84.4	0.773	0.773	0.886
'colsample_bytree': 0.7, 'eta': 0.2, 'max_depth': 10, 'min_child_weight': 0.4, 'subsample': 0.7, 'tree_method': 'hist', 'objective':'binary:logistic'	MC (0.75)	26	95.5	93.8	0.910	0.910	0.955
			91.4	88.3	0.828	0.827	0.914
'colsample_bytree': 0.8, 'eta': 0.1, 'max_depth': 10, 'min_child_weight': 1, 'subsample': 0.7, 'tree_method': 'hist', 'objective':'binary:logistic'	RFECV	24	95.4	93.3	0.907	0.907	0.954
			90.5	86.7	0.809	0.809	0.905
HemoPI-2 model and validation datasets							
'colsample_bytree': 0.8, 'eta': 0.1, 'max_depth': 10, 'min_child_weight': 0.8, 'subsample': 0.7, 'tree_method': 'approx', 'objective':'binary:logistic'	none	56	77.8	74.3	0.552	0.552	0.776
			67.3	64.8	0.337	0.335	0.666
'colsample_bytree': 1.0, 'eta': 0.3, 'max_depth': 10, 'min_child_weight': 1, 'subsample': 0.9, 'tree_method': 'hist', 'objective':'binary:logistic'	MC (0.75)	27	77.5	73.8	0.545	0.545	0.771
			69.8	66.8	0.388	0.386	0.691
'colsample_bytree': 0.8, 'eta': 0.1, 'max_depth': 14, 'min_child_weight': 1, 'subsample': 0.7, 'tree_method': 'hist', 'objective':'binary:logistic'	RFECV	34	79.1	75.3	0.577	0.577	0.787
			70.3	67.2	0.398	0.397	0.697
HemoPI-3 model and validation datasets							
'colsample_bytree': 0.7, 'eta': 0.1, 'max_depth': 10, 'min_child_weight': 0.4, 'subsample': 0.7, 'tree_method': 'hist', 'objective':'binary:logistic'	none	56	78.3	74.6	0.561	0.560	0.779
			70.8	67.5	0.408	0.406	0.701
'colsample_bytree': 0.8, 'eta': 0.2, 'max_depth': 14, 'min_child_weight': 0.2, 'subsample': 0.8, 'tree_method': 'approx', 'objective':'binary:logistic'	MC (0.75)	28	78.7	74.9	0.569	0.568	0.783
			72.6	69.1	0.445	0.444	0.720
'colsample_bytree': 0.8, 'eta': 0.1, 'max_depth': 10, 'min_child_weight': 0.4, 'subsample': 0.7, 'tree_method': 'hist', 'objective':'binary:logistic'	RFECV	21	78.2	74.3	0.559	0.558	0.777
			70.2	67.6	0.398	0.398	0.699

Table S5b. Performance metrics of binary classifier Extreme Gradient Boosting for HemoPI datasets with full or reduced number of features (multicollinearity, recursive feature elimination) using optimized model hyperparameters.

**Model datasets**	Nb. Peptides	Novelty	Inlier	Fraction
HemoPI-1	884	47	837	0.05
HemoPI-2	812	23	789	0.03
HemoPI-3	1298	50	1248	0.04
**APD datasets**	Nb. Peptides	Outlier	Inlier	Fraction
APD / HemoPI-1	3081	337	2744	0.11
APD / HemoPI-2	3081	440	2641	0.14
APD / HemoPI-3	3081	89	2992	0.03
**HAMP datasets**	Nb. Peptides	Novelty	Inlier	Fraction
HAMP / HemoPI-1	317	16	301	0.05
HAMP / HemoPI-2	317	52	265	0.16
HAMP / HemoPI-3	317	4	313	0.01

Table S6. Inliers, novelties (model datasets) and outliers (APD and HAMP datasets) counts applying empirical rule to Mahalanobis distances for HemoPI-1, HemoPI-2, HemoPI-3 datasets.

Outliers fraction	0.05		0.03		0.04	
	**HemoPI-1**		**HemoPI-2**		**HemoPI-3**	
Nb. peptides	884		812		1298	
Detection method	Novelty	Inlier	Novelty	Inlier	Novelty	Inlier
ABOD	63	821	35	777	70	1228
CBLOF	45	839	25	787	52	1246
FB	36	848	21	791	42	1256
HBOS	45	839	25	787	52	1246
IF	45	839	25	787	52	1246
KNN	31	853	21	791	40	1258
Average KNN	**14**	**870**	**5**	**807**	**10**	**1288**
LOF	31	853	19	793	42	1256
	**APD/HemoPI-1**		**APD/HemoPI-2**		**APD/HemoPI-3**	
Nb. peptides	3081		3081		3081	
Detection method	Outlier	Inlier	Outlier	Inlier	Outlier	Inlier
ABOD	337	2744	376	2705	309	2772
CBLOF	278	2803	307	2774	354	2727
FB	275	2806	195	2886	221	2860
HBOS	157	2924	108	2973	118	2963
IF	180	2901	100	2981	170	2911
KNN	262	2819	205	2876	222	2859
Average KNN	**273**	**2808**	**264**	**2817**	**253**	**2828**
LOF	267	2814	210	2871	452	2629
	**HAMP/HemoPI-1**		**HAMP/HemoPI-2**		**HAMP/HemoPI-3**	
Nb. peptides	317		317		317	
Detection method	Novelty	Inlier	Novelty	Inlier	Novelty	Inlier
ABOD	4	313	6	311	7	310
CBLOF	13	304	13	304	38	279
FB	3	314	1	316	3	314
HBOS	7	310	0	317	3	314
IF	4	313	0	317	7	310
KNN	5	312	1	316	3	314
Average KNN	**3**	**314**	**3**	**314**	**3**	**314**
LOF	4	313	2	315	12	305

Table S7. Inliers, novelties and outliers counts applying multivariate outlier detection methods; ABOD: Angle-based outlier detection, CBLOF: Cluster-based local outlier factor, FB: Feature bagging, HBOS: Histogram-based outlier detection, IF: Isolation forest, (Average) KNN: K-nearest neighbors detector, LOF: Local outlier factor to 56-dimensional datasets HemoPI-1, HemoPI-2, HemoPI-3, Antimicrobial Peptide Dataset (APD, 3081) and known haemolytic antimicrobial peptides in APD (HAMP, 317). – not reported.

	Physicochemical property	Test	p-value	p-adjust
1	H_Eisenberg	Welch	4.29×10^{-6}	5.34×10^{-6}
2	uH_Eisenberg	Wilcoxon	5.39×10^{-13}	1.59×10^{-12}
3	modlas_ABHPRK	Wilcoxon	8.18×10^{-1}	8.18×10^{-1}
4	uB_Bulkiness	Wilcoxon	1.95×10^{-3}	2.02×10^{-3}
5	uH_HoppWoods	Wilcoxon	3.87×10^{-12}	9.02×10^{-12}
6	u_polarity	Wilcoxon	1.74×10^{-9}	2.86×10^{-9}
7	H_GRAVY	Kolmogorov-Smirnov	0.00	0.00
8	uH_GRAVY	Kolmogorov-Smirnov	1.67×10^{-13}	5.19×10^{-13}
9	Z3_1	Kolmogorov-Smirnov	5.21×10^{-10}	9.42×10^{-10}
10	Z3_2	Kolmogorov-Smirnov	1.31×10^{-12}	3.40×10^{-12}
11	Z3_3	Kolmogorov-Smirnov	4.93×10^{-8}	6.90×10^{-8}
12	Z5_1	Kolmogorov-Smirnov	1.93×10^{-4}	2.19×10^{-4}
13	Z5_2	Kolmogorov-Smirnov	1.11×10^{-16}	6.22×10^{-16}
14	Z5_3	Kolmogorov-Smirnov	1.13×10^{-11}	2.35×10^{-11}
15	Z5_4	Kolmogorov-Smirnov	4.07×10^{-9}	6.16×10^{-9}
16	Z5_5	Kolmogorov-Smirnov	3.86×10^{-2}	3.93×10^{-2}
17	S_AASI	Kolmogorov-Smirnov	1.31×10^{-7}	1.70×10^{-7}
18	uS_AASI	Kolmogorov-Smirnov	2.87×10^{-9}	4.59×10^{-9}
19	H_argos	Kolmogorov-Smirnov	0.00	0.00
20	uH_argos	Kolmogorov-Smirnov	0.00	0.00
21	B_Bulkiness	Kolmogorov-Smirnov	2.05×10^{-14}	7.67×10^{-14}
22	charge_phys	Kolmogorov-Smirnov	3.35×10^{-12}	8.17×10^{-12}
23	charge_acid	Kolmogorov-Smirnov	3.06×10^{-10}	5.92×10^{-10}
24	Ez	Kolmogorov-Smirnov	1.96×10^{-4}	2.19×10^{-4}
25	flexibility	Kolmogorov-Smirnov	0.00	0.00
26	u_flexbility	Kolmogorov-Smirnov	1.03×10^{-7}	1.38×10^{-7}
27	Grantham	Kolmogorov-Smirnov	7.57×10^{-12}	1.63×10^{-11}
28	H_HoppWoods	Kolmogorov-Smirnov	1.18×10^{-4}	1.41×10^{-4}
29	ISAECI	Kolmogorov-Smirnov	5.95×10^{-14}	2.08×10^{-13}
30	H_Janin	Kolmogorov-Smirnov	1.33×10^{-5}	1.61×10^{-5}
31	uH_Janin	Kolmogorov-Smirnov	1.78×10^{-15}	7.65×10^{-15}
32	H_KyteDoolittle	Kolmogorov-Smirnov	0.00	0.00
33	uH_KyteDoolittle	Kolmogorov-Smirnov	1.34×10^{-12}	3.40×10^{-12}
34	F_Levitt	Kolmogorov-Smirnov	0.00	0.00
35	uF_Levitt	Kolmogorov-Smirnov	4.97×10^{-4}	5.35×10^{-4}
36	MSS_shape	Kolmogorov-Smirnov	3.74×10^{-8}	5.38×10^{-8}
37	u_MSS_shape	Kolmogorov-Smirnov	2.17×10^{-4}	2.39×10^{-4}
38	MSW	Kolmogorov-Smirnov	3.78×10^{-7}	4.82×10^{-7}
39	pepArc	Kolmogorov-Smirnov	3.18×10^{-10}	5.93×10^{-10}
40	pepcats	Kolmogorov-Smirnov	1.21×10^{-4}	1.41×10^{-4}
41	polarity	Kolmogorov-Smirnov	1.57×10^{-10}	3.15×10^{-10}
42	PPCALI	Kolmogorov-Smirnov	9.68×10^{-10}	1.64×10^{-9}
43	refractivity	Kolmogorov-Smirnov	4.51×10^{-9}	6.64×10^{-9}
44	u_refractivity	Kolmogorov-Smirnov	6.14×10^{-4}	6.48×10^{-4}
45	t_scale	Kolmogorov-Smirnov	5.55×10^{-16}	2.83×10^{-15}
46	TM_tend	Kolmogorov-Smirnov	4.04×10^{-9}	6.16×10^{-9}

Table S9. Statistical values (p-value and p-adjust) for all 56 physicochemical properties.

	Physicochemical property	Test	p-value	p-adjust
47	u_TM_tend	Kolmogorov-Smirnov	6.77×10^{-15}	2.71×10^{-14}
48	Length	Kolmogorov-Smirnov	0.00	0.00
49	BomanIndex	Kolmogorov-Smirnov	7.13×10^{-14}	2.35×10^{-13}
50	Aromaticity	Kolmogorov-Smirnov	5.44×10^{-8}	7.42×10^{-8}
51	AliphaticIndex	Kolmogorov-Smirnov	0.00	0.00
52	InstabilityIndex	Kolmogorov-Smirnov	7.43×10^{-13}	2.08×10^{-12}
53	NetCharge	Kolmogorov-Smirnov	6.08×10^{-12}	1.36×10^{-11}
54	MW	Kolmogorov-Smirnov	1.11×10^{-15}	5.18×10^{-15}
55	IsoelectricPoint	Kolmogorov-Smirnov	6.10×10^{-10}	1.07×10^{-9}
56	HydrophobicRatio	Kolmogorov-Smirnov	0.00	0.00

Table S9 (suite). Statistical values (p-value and p-adjust) for all 56 physicochemical properties.

Physicochemical property	APD inliers (2,808)					
	minimum	1st quartile	median	mean	3rd quartile	maximum
H_Eisenberg	-1.12	-0.31	-0.01	-0.02	0.28	1.02
uH_Eisenberg	0.00	0.07	0.14	0.21	0.29	1.15
modlas_ABHPRK	-3.50	-1.14	-0.60	-0.45	0.29	2.93
uB_Bulkiness	0.00	0.19	0.43	0.60	0.83	2.80
uH_HoppWoods	1.58	5.87	7.34	8.02	9.07	18.28
u_polarity	0.61	3.96	6.25	7.18	8.68	21.74
H_GRAVY	0.30	1.64	3.32	3.50	5.11	10.01
uH_GRAVY	1.80	6.27	7.57	8.09	9.18	16.16
Z3_1	0.71	3.62	5.11	5.60	6.95	13.04
Z3_2	0.11	1.67	3.00	3.28	4.44	9.34
Z3_3	0.26	1.35	2.01	2.42	2.83	9.64
Z5_1	0.09	0.69	1.02	1.18	1.64	3.24
Z5_2	0.93	1.95	2.13	2.07	2.26	3.13
Z5_3	0.00	0.05	0.10	0.18	0.26	1.66
Z5_4	0.00	0.00	1.00	0.63	1.00	2.00
Z5_5	-1.00	-0.27	-0.10	-0.04	0.11	1.49
S_AASI	0.00	0.07	0.11	0.19	0.21	1.32
uS_AASI	0.19	0.51	0.60	0.59	0.70	0.91
H_argos	0.00	0.03	0.05	0.07	0.10	0.58
uH_argos	-1.00	0.00	0.07	0.08	0.19	0.58
B_Bulkiness	-1.00	0.00	0.10	0.11	0.21	0.62
charge_phys	15.97	18.75	19.55	19.67	20.50	24.42
charge_acid	0.15	0.54	0.62	0.62	0.72	0.89
Ez	0.00	0.03	0.05	0.08	0.11	0.42
flexibility	33.03	69.71	81.68	82.89	92.64	153.10
u_flexbility	-2.05	-0.43	-0.08	-0.05	0.27	3.00
Grantham	0.00	0.12	0.25	0.37	0.48	1.88
H_HoppWoods	19.71	69.12	79.62	82.81	97.93	147.91
ISAECI	-1.07	-0.24	0.02	0.00	0.32	0.89
H_Janin	0.00	0.07	0.14	0.19	0.28	0.91
uH_Janin	-1.00	-0.21	-0.03	0.02	0.27	1.15
H_KyteDoolittle	0.00	0.06	0.14	0.20	0.27	0.93
uH_KyteDoolittle	0.62	0.85	0.96	0.94	1.02	1.37
F_Levitt	0.00	0.03	0.05	0.08	0.12	0.88
uF_Levitt	6.35	15.87	18.70	17.54	19.82	23.13
MSS_shape	0.00	0.63	1.16	1.78	2.45	14.21
u_MSS_shape	-1.85	-0.51	-0.25	-0.24	-0.02	1.25
MSW	0.00	0.00	1.00	0.65	1.00	2.00
pepArc	0.00	1.00	1.00	1.29	2.00	3.00
pepcats	0.08	0.35	0.42	0.42	0.48	1.00
polarity	0.00	0.03	0.05	0.07	0.10	0.46
PPCALI	-5.28	-1.76	-0.83	-0.73	0.01	3.57
refractivity	0.14	0.30	0.39	0.39	0.46	0.79
u_refractivity	0.00	0.02	0.04	0.05	0.08	0.40
t_scale	-37.03	-15.92	-8.54	-8.40	-2.90	26.86
TM_tend	-3.27	-0.86	-0.50	-0.47	-0.05	1.50

Table S10. Descriptive statistics for 56 physicochemical property distributions (APD inliers).

Physicochemical property	APD inliers (2,808)					
	minimum	1st quartile	median	mean	3rd quartile	maximum
u_TM_tend	0.00	0.12	0.23	0.35	0.47	1.84
Length	2.00	10.00	22.00	44.34	63.75	183.00
BomanIndex	-3.82	0.17	1.56	1.65	2.82	8.72
Aromaticity	0.00	0.00	0.08	0.11	0.17	0.50
AliphaticIndex	0.00	21.14	51.70	65.53	83.04	292.50
InstabilityIndex	-43.43	15.01	38.06	47.96	67.51	295.57
NetCharge	-8.97	-0.01	1.99	3.35	5.96	29.99
MW	260.29	1243.64	2378.53	4760.15	6638.02	19719.83
IsoelectricPoint	2.43	6.00	8.90	8.66	10.98	13.53
HydrophobicRatio	0.00	0.17	0.30	0.31	0.42	0.88

Table S10 (suite). Descriptive statistics for 56 physicochemical property distributions (inliers).

Physicochemical property	APD outliers (273)					
	minimum	1st quartile	median	mean	3rd quartile	maximum
H_Eisenberg	-0.89	-0.08	0.11	0.10	0.29	0.84
uH_Eisenberg	0.00	0.13	0.23	0.26	0.37	0.94
modlas_ABHPRK	-2.66	-0.40	0.14	0.15	0.68	2.17
uB_Bulkiness	0.01	0.38	0.71	0.82	1.21	2.44
uH_HoppWoods	2.96	7.06	8.25	8.49	9.69	14.75
u_polarity	1.39	4.30	5.48	5.55	6.69	14.32
H_GRAVY	0.47	2.39	3.44	3.54	4.61	10.12
uH_GRAVY	2.81	6.82	7.74	7.96	8.88	13.76
Z3_1	1.42	3.84	4.54	4.59	5.36	9.35
Z3_2	0.81	2.32	3.11	3.27	4.08	8.98
Z3_3	0.47	1.31	1.76	1.85	2.27	6.59
Z5_1	0.14	0.67	0.99	1.12	1.55	3.15
Z5_2	1.47	2.00	2.11	2.09	2.20	2.67
Z5_3	0.00	0.07	0.12	0.13	0.17	0.55
Z5_4	0.00	0.00	1.00	0.63	1.00	1.00
Z5_5	-0.66	-0.08	0.07	0.12	0.28	1.23
S_AASI	0.01	0.11	0.22	0.27	0.41	1.05
uS_AASI	0.41	0.58	0.62	0.63	0.67	0.86
H_argos	0.00	0.03	0.06	0.08	0.10	0.30
uH_argos	-0.27	0.05	0.10	0.11	0.16	0.60
B_Bulkiness	-0.27	0.07	0.12	0.13	0.17	0.60
charge_phys	16.31	19.11	19.62	19.71	20.19	23.41
charge_acid	0.36	0.53	0.57	0.57	0.61	0.86
Ez	0.00	0.04	0.07	0.07	0.10	0.25
flexibility	50.46	78.96	84.62	85.48	91.13	132.55
u_flexbility	-1.37	-0.41	-0.14	-0.15	0.10	1.40
Grantham	0.00	0.22	0.40	0.44	0.63	1.58
H_HoppWoods	51.79	79.25	85.95	88.70	97.05	138.16
ISAECI	-1.29	-0.09	0.06	0.06	0.25	0.69
H_Janin	0.00	0.13	0.24	0.26	0.38	1.04
uH_Janin	-0.72	0.04	0.22	0.22	0.40	0.89
H_KyteDoolittle	0.01	0.13	0.24	0.27	0.40	0.79
uH_KyteDoolittle	0.79	0.97	1.01	1.01	1.05	1.24
F_Levitt	0.00	0.03	0.05	0.07	0.09	0.27
uF_Levitt	13.01	17.31	18.35	18.18	19.20	22.09
MSS_shape	0.02	0.72	1.19	1.41	1.84	5.71
u_MSS_shape	-0.88	-0.48	-0.34	-0.31	-0.18	0.75
MSW	0.00	1.00	1.00	0.86	1.00	1.00
pepArc	0.00	1.00	1.00	1.21	1.00	2.00
pepcats	0.18	0.35	0.40	0.39	0.44	0.64
polarity	0.00	0.04	0.07	0.08	0.12	0.25
PPCALI	-3.38	-1.00	-0.36	-0.38	0.25	3.18
refractivity	0.20	0.34	0.39	0.39	0.43	0.67
u_refractivity	0.00	0.02	0.04	0.05	0.06	0.21
t_scale	-23.68	-10.24	-7.58	-7.39	-4.70	9.34
TM_tend	-1.78	-0.58	-0.34	-0.30	-0.04	1.02

Table S11. Descriptive statistics for 56 physicochemical property distributions (APD outliers).

Physicochemical property	APD outliers (273)					
	minimum	1st quartile	median	mean	3rd quartile	maximum
u_TM_tend	0.01	0.22	0.41	0.48	0.70	1.69
Length	7.00	21.00	29.00	31.82	38.00	131.00
BomanIndex	-3.13	-0.22	0.73	0.78	1.78	6.39
Aromaticity	0.00	0.04	0.08	0.08	0.12	0.39
AliphaticIndex	0.00	57.78	91.52	93.33	122.00	240.00
InstabilityIndex	-38.59	8.76	24.12	26.98	41.52	165.30
NetCharge	-11.85	0.99	2.68	2.95	4.68	19.02
MW	763.82	2206.64	3052.54	3476.07	4200.31	14700.56
IsoelectricPoint	2.65	8.34	10.03	9.43	10.71	13.06
HydrophobicRatio	0.03	0.35	0.43	0.44	0.52	0.75

Table S11 (suite). Descriptive statistics for 56 physicochemical property distributions (outliers).

Amino acid properties		RPS inliers		RPS outliers
		**Quadrant 1 % (mean, stdev)**	**Quadrant 4 % (mean, stdev)**	**Quadrant 5 % (mean, stdev)**
charged	K / R / H	15.1 ± 4.3	**23.8 ± 5.1**	16.9 ± 9.1
	D / E	**6.5 ± 3.2**	2.5 ± 2.6	3.4 ± 3.8
small	G / P	12.2 ± 4.7	12.1 ± 5.2	**19.3 ± 7.5**
	G / C / A / P / S	29.6 ± 6.0	30.4 ± 7.2	**42.3 ± 9.1**
bulky	L / I	**19.9 ± 5.9**	18.4 ± 6.6	15.6 ± 7.8
	F / W / Y	**10.4 ± 4.9**	10.2 ± 5.1	6.9 ± 5.2
	F / L / I / W / Y	**30.4 ± 6.0**	28.6 ± 6.9	22.5 ± 7.5
Global percentage		81.6 ± 17.5	85.3 ± 10.4	85.1 ± 14.8

Table S12. Amino acid composition for specific random peptide sequences (RPS), expressed in percentages (means, stdev: standard deviation).

Amino acid properties		APD (3,081) % (mean, stdev)	RPS (5,000) % (mean, stdev)
charged	K / R / H	17.4 ± 9.4	17.9 ± 7.1
	D / E	4.5 ± 5.9	4.1 ± 3.7
small	G / P	15.7 ± 9.4	15.0 ± 6.6
	G / C / A / P / S	35.7 ± 12.2	34.9 ± 9.0
bulky	L / I	16.9 ± 11.5	18.1 ± 7.2
	F / W / Y	8.5 ± 6.6	8.9 ± 5.3
	F / L / I / W / Y	25.5 ± 12.1	27.1 ± 8.3
Global percentage		83.1 ± 19.8	84.0 ± 14.0

Table S13. Amino acid compositions for APD and RPS datasets, expressed in percentages (means, stdev: standard deviation).

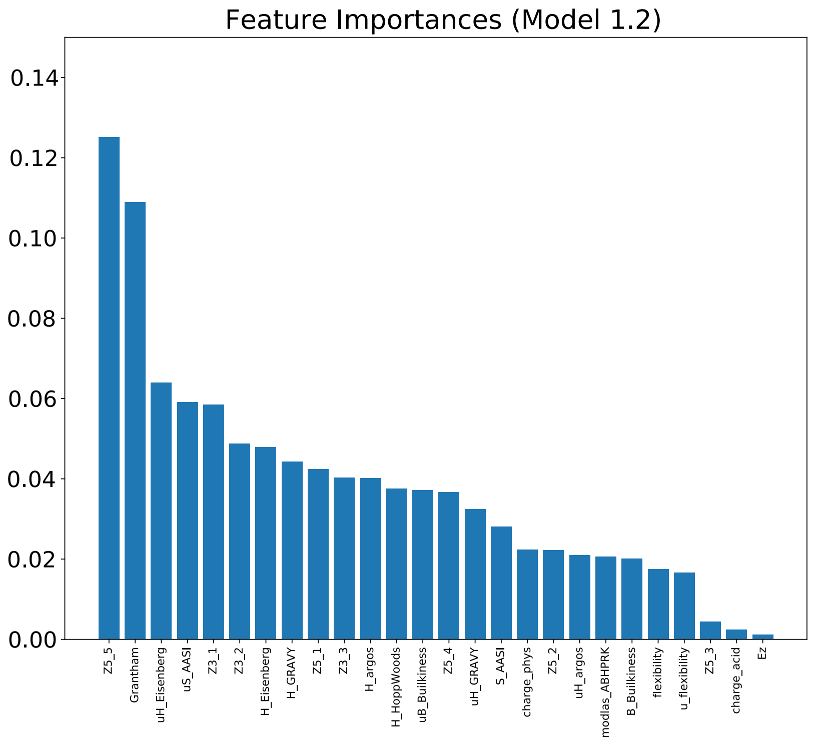

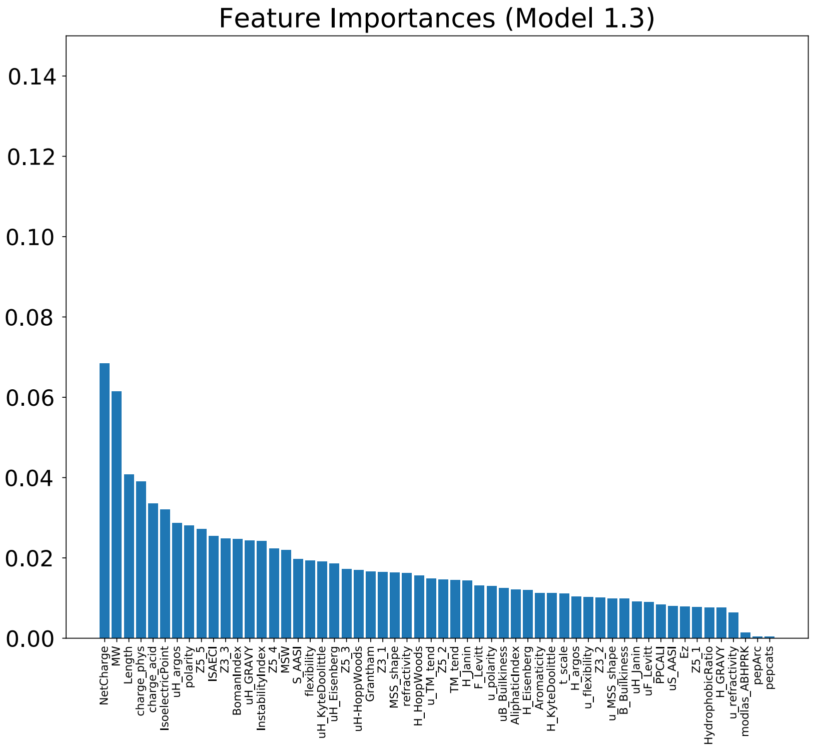

Figure S1a. Feature importances of top 2 binary classifiers for HemoPI-1 dataset.

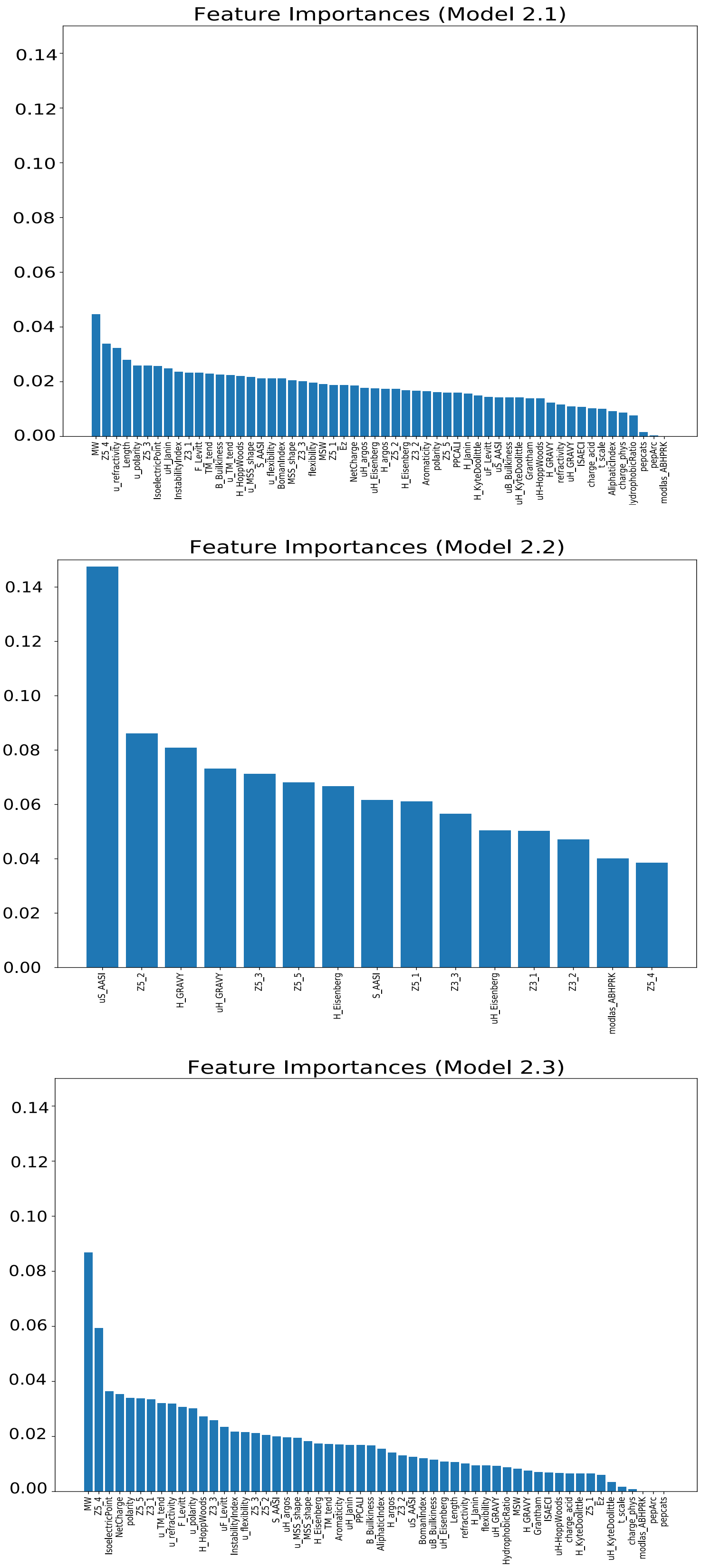

Figure S1b. Feature importances of top 3 binary classifiers for HemoPI-2 dataset.

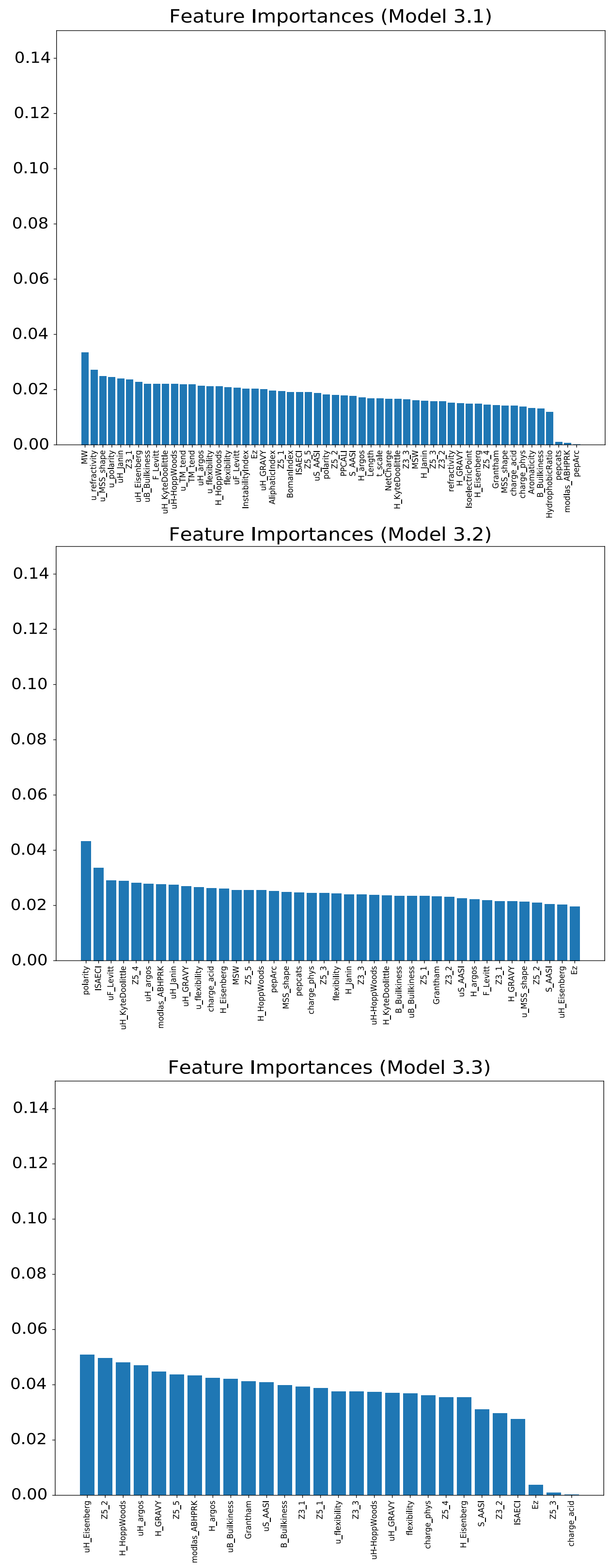

Figure S1c. Feature importances of top 3 binary classifiers for HemoPI-3 dataset.

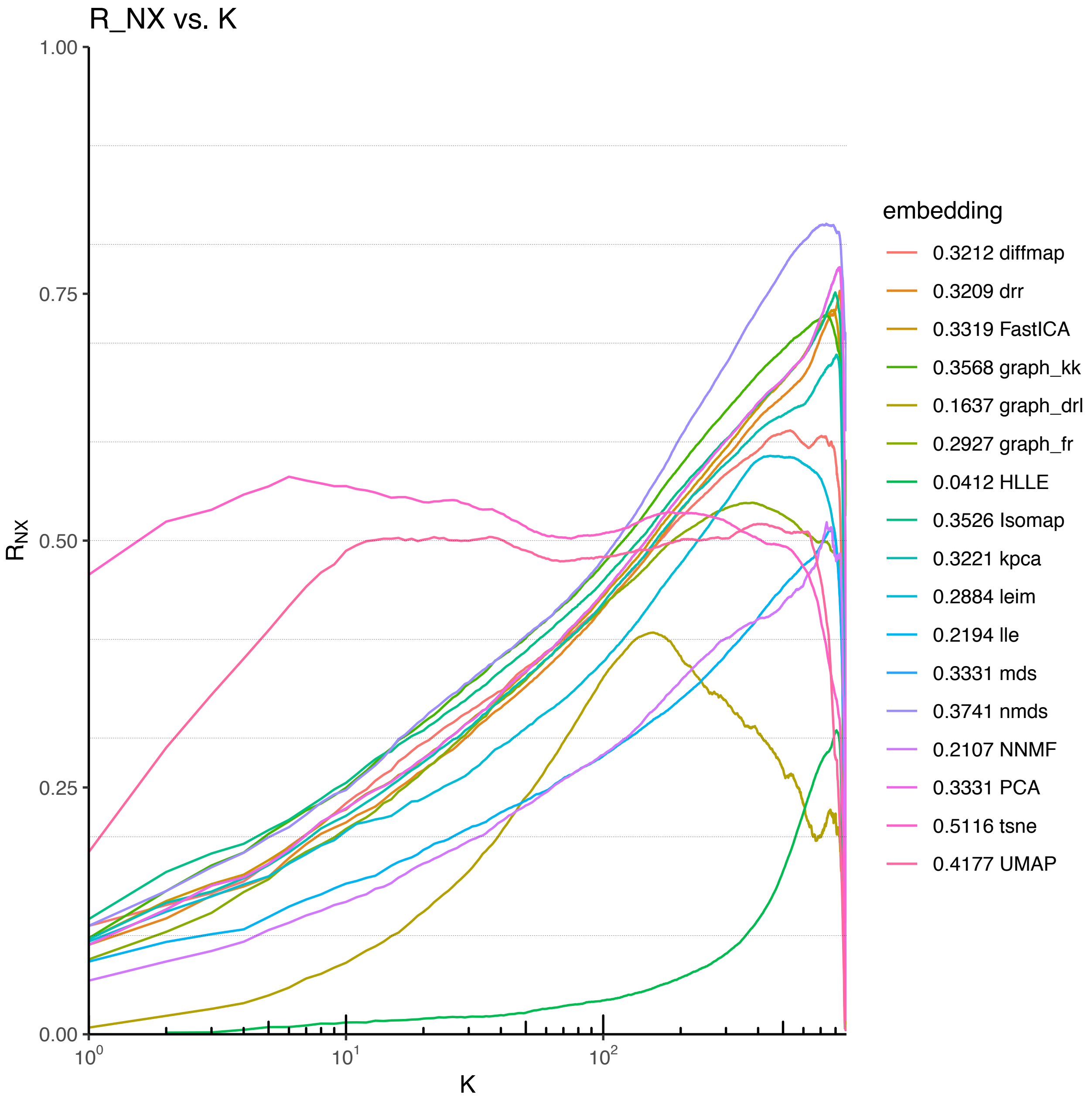

Figure S2. Quality curves $R_{NX}(K)$ report the relative improvement over 17 different embedding techniques for HemoPI-1 model dataset. The higher the curve, the better AUCs stand in the legend. Embeddings are: diffmap (Diffusion maps), drr (Dimensionality reduction via regression), FastICA (Independent Component Analysis), graph_kk (Graph embedding via Kamada Kawai algorithm), graph_drl (Distributed recursive graph layout), graph_fr (Fruchterman Reingold graph layout), HLLE (Hessian Locally Linear Embedding), Isomap (Isomap embedding), kPCA (kernel Principal Component Analysis), LEIM (Laplacian Eigenmaps), LLE (Locally Linear Embedding), MDS (Multidimensional Scaling), NMDS (Non-metric Multidimensional Scaling), NNMF (Non-Negative Matrix Factorization), PCA (Principal Component Analysis), t-SNE (t-distributed stochastic neighbour embedding) and UMAP (Uniform Manifold Approximation).

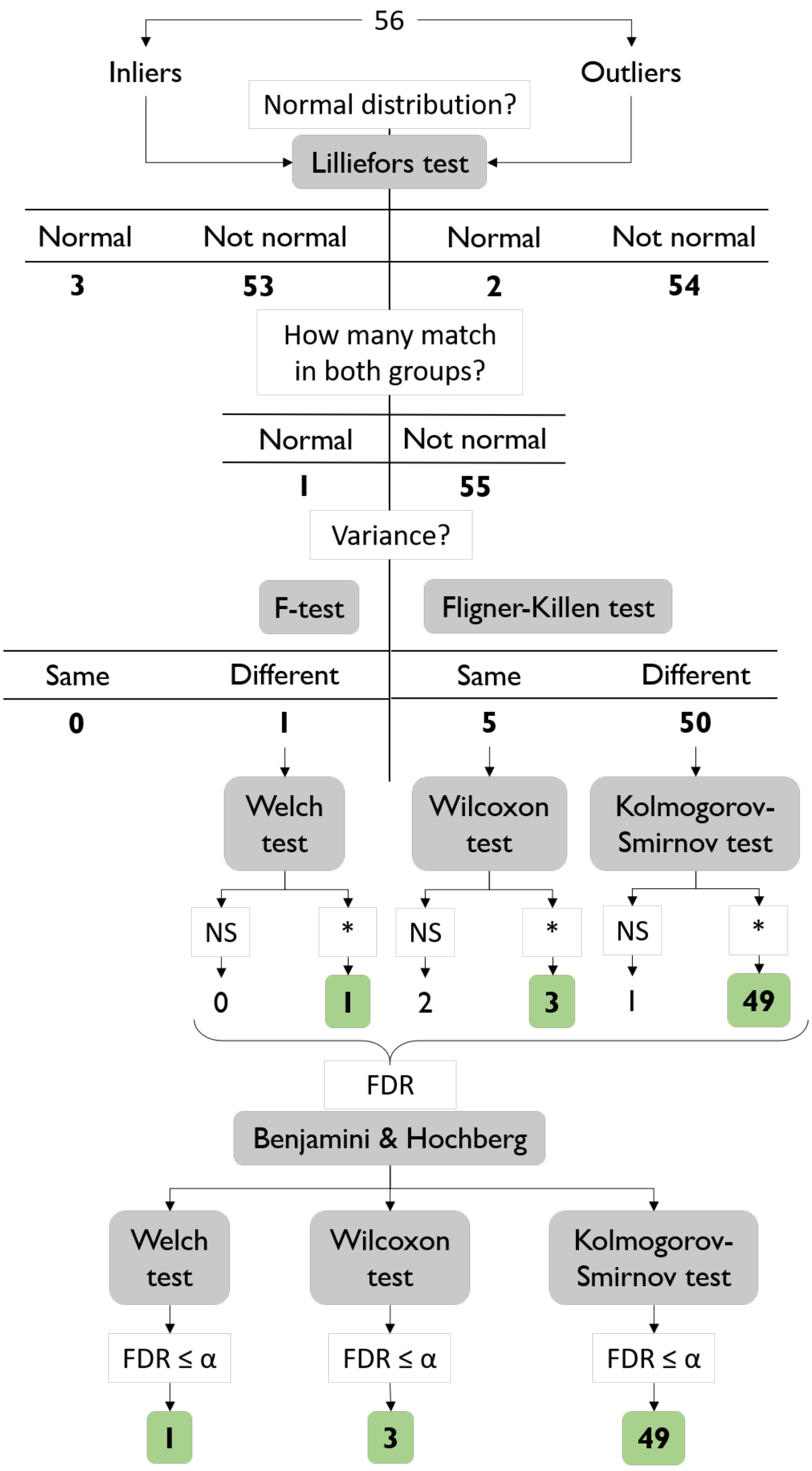

Figure S3. Statistical studies to identify differences between the distributions of 56 physicochemical properties from two groups; APD inliers and outliers.

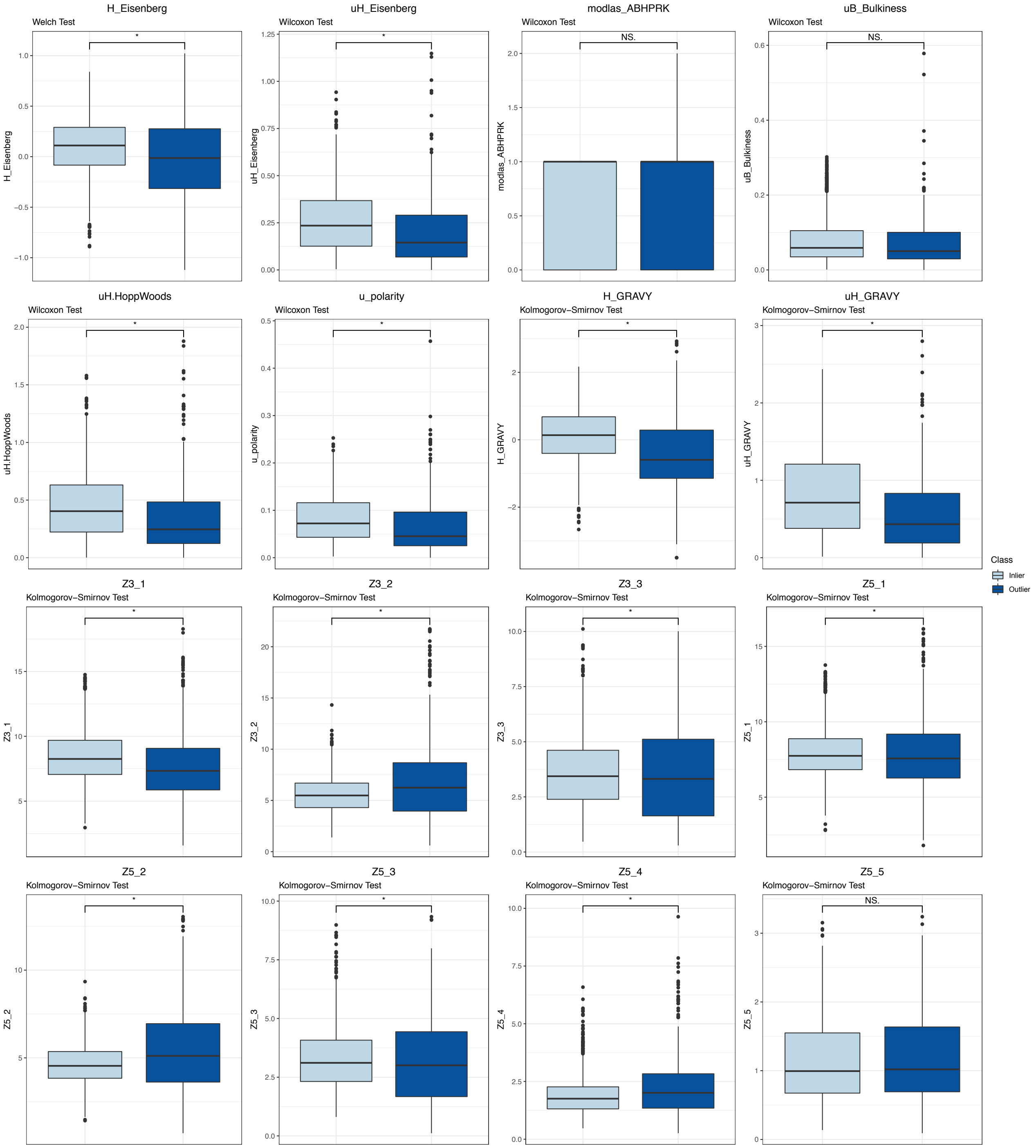

Figure S4a. Boxplots comparing the distributions for each of the first 16 physicochemical properties (1-16: H_Eisenberg to Z5_5) within inliers (cyan) and outliers (dark blue) groups. All properties were tested for statistical significance (*, p-value <0.001) using either Welch, Wilcoxon or Kolmogorov-Smirnov tests according to normality and variance.

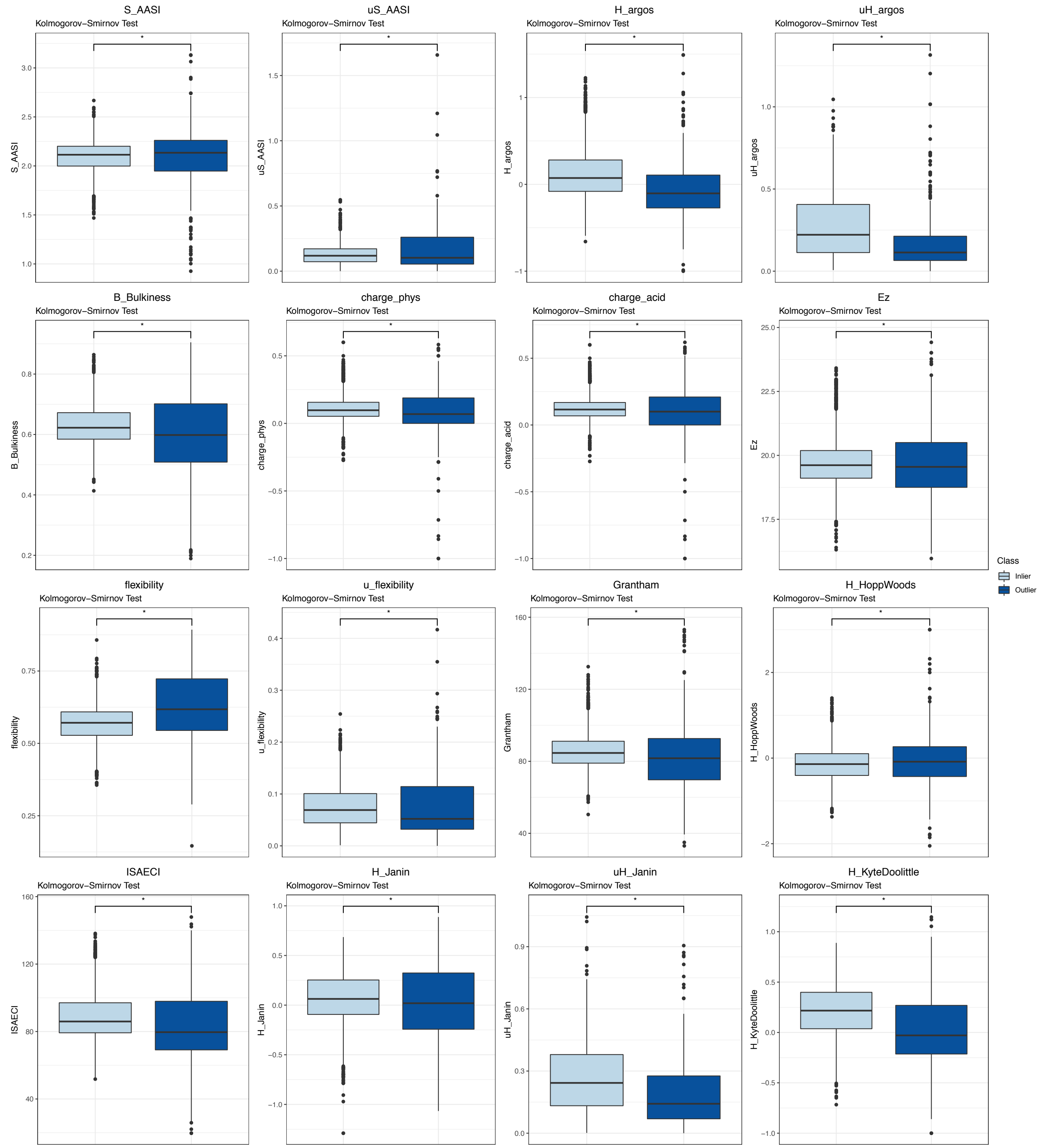

Figure S4b. Boxplots comparing the distributions for each of the 16 other physicochemical properties (17-32: S_AASI to H_KyteDoolittle) within inliers (cyan) and outliers (dark blue) groups. All properties were tested for statistical significance (*, p-value <0.001) using either Welch, Wilcoxon or Kolmogorov-Smirnov tests according to normality and variance.

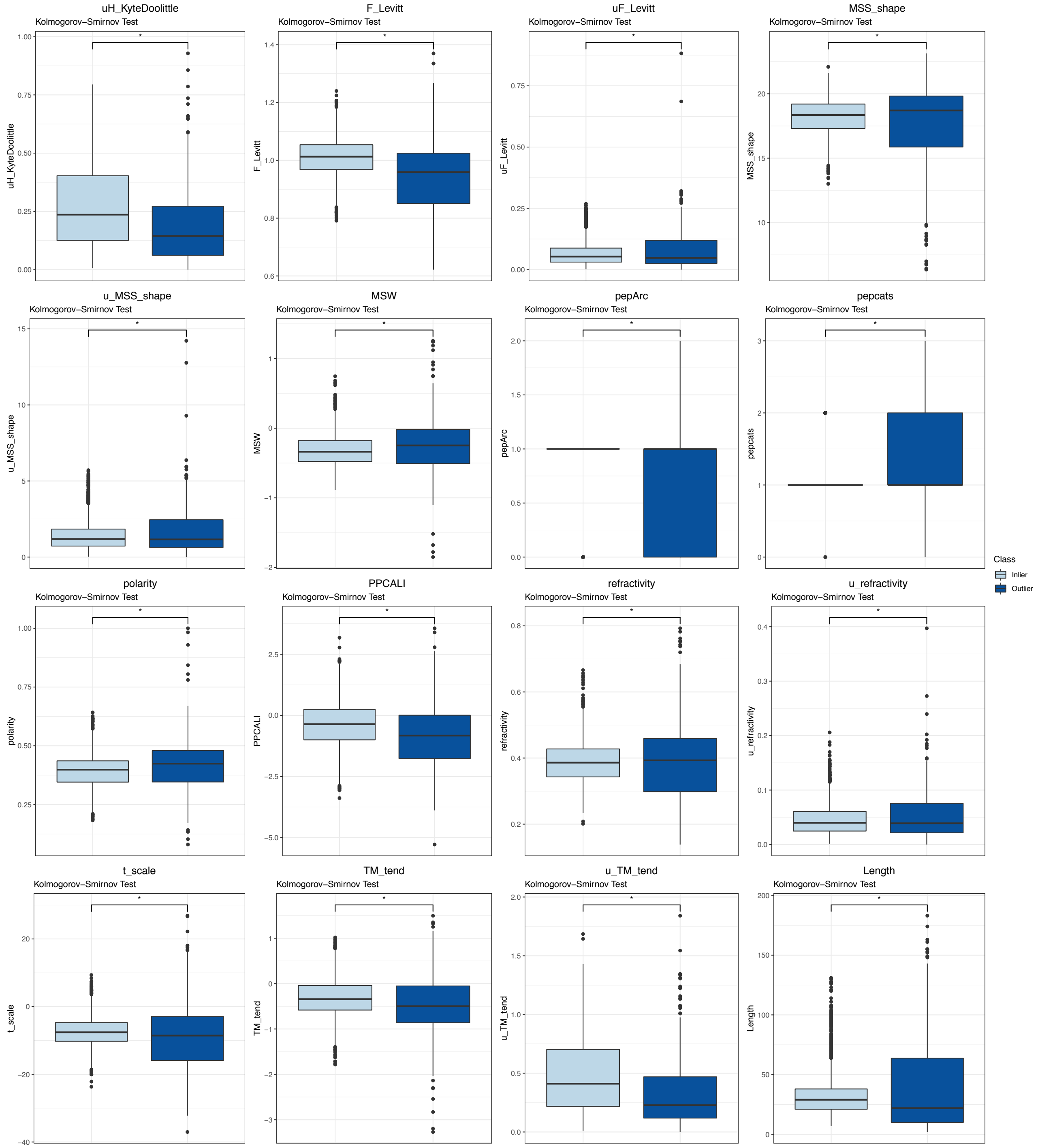

Figure S4c. Boxplots comparing the distributions for each of the 16 physicochemical properties (32-48: uH_KyteDoolittle to Length) within inliers (cyan) and outliers (dark blue) groups. All properties were tested for statistical significance (*, p-value <0.001) using either Welch, Wilcoxon or Kolmogorov-Smirnov tests according to normality and variance.

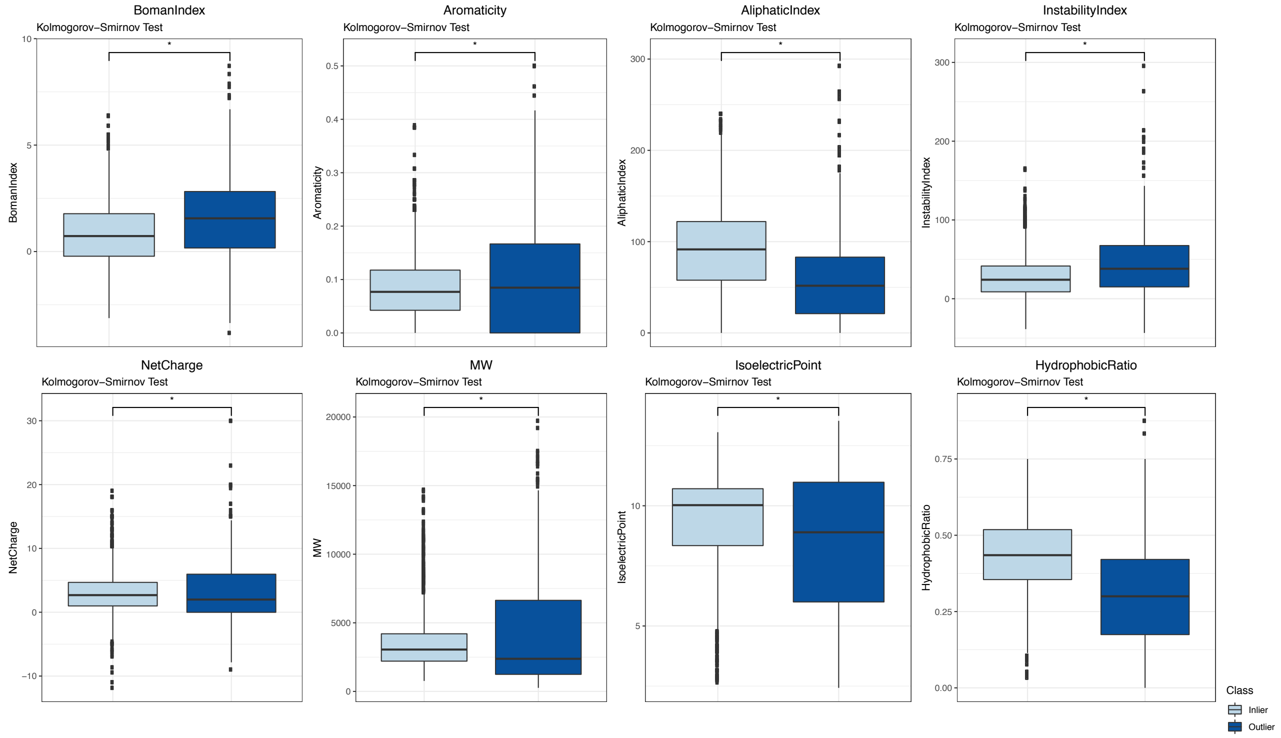

Figure S4d. Boxplots comparing the distributions for each of the 16 physicochemical properties (49-56: BomanIndex to HydrophobicRatio) within inliers (cyan) and outliers (dark blue) groups. All properties were tested for statistical significance (*, p-value <0.001) using either Welch, Wilcoxon or Kolmogorov-Smirnov tests according to normality and variance.

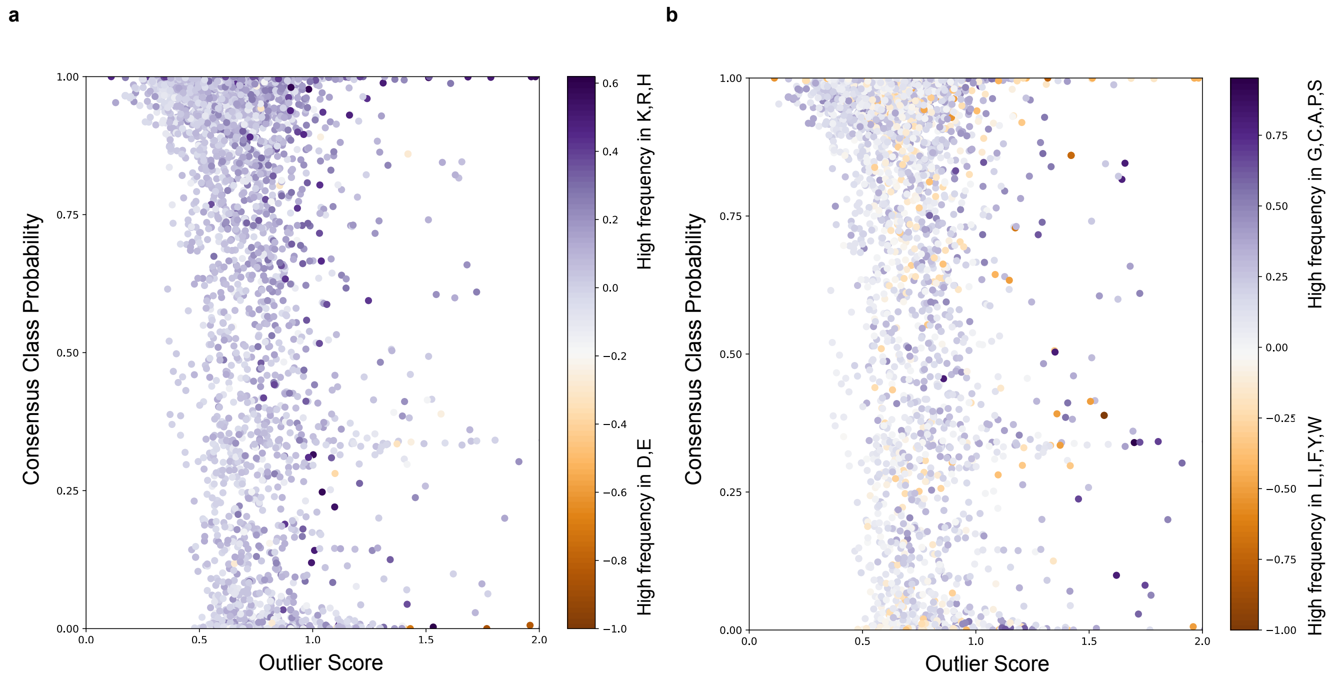

Figure S5. Scatterplots showing the distribution of 3,081 antimicrobial peptides (APD dataset) according to outlier scores and hemolytic (HemoPI-1) consensus class probabilities: a) Changes in colour gradient indicate differential enrichment in positively charged amino acids i.e. lysine, arginine, histidine (purple) or negatively charged amino acids i.e. aspartic and glutamic acids (orange). b) Changes in colour gradient indicate differential enrichment in small amino acids i.e. glycine, cysteine, alanine, proline, serine (purple) or bulky aromatic/aliphatic amino acids i.e. phenylalanine, tyrosine, tryptophan, leucine, isoleucine (orange).
